# Gene Signature-Based Drug Screening Reveals Ponatinib Enhances Immunotherapy Efficacy in Triple-Negative Breast Cancer by Reversing MDSC-Mediated Immunosuppressive Tumor Microenvironment

**DOI:** 10.34133/research.0915

**Published:** 2025-10-09

**Authors:** Qianyu Wang, Shasha Li, Yuan Wu, Xiankuo Yu, Yifei Dai, Yumei Wang, Lu Li, Ming Yang, Kequan Lin, Wei Shao, Haiyan Wang, Huili Wang, Guanbin Zhang, Dong Wang

**Affiliations:** ^1^ State Key Laboratory of Southwestern Chinese Medicine Resources, School of Basic Medical Sciences, Chengdu University of Traditional Chinese Medicine, Chengdu 611137, China.; ^2^ School of Life Sciences, Tsinghua University, Beijing 100084, China.; ^3^ Department of Endocrinology and Metabolism, Guangdong Provincial Key Laboratory of Diabetology, The Third Affiliated Hospital of Sun Yat-Sen University, Guangzhou 510000, China.; ^4^ Department of Basic Medical Sciences, School of Medicine, Tsinghua University, Beijing 100084, China.; ^5^ Department of Statistics and Data Science, Tsinghua University, Beijing 100084, China.; ^6^ Department of Cardiology of The Second Affiliated Hospital, School of Medicine, Zhejiang University, Hangzhou 310009, China.; ^7^ Cardiovascular Key Laboratory of Zhejiang Province, Hangzhou 310009, China.; ^8^ Chinese Institutes for Medical Research, Beijing 100069, China.; ^9^ Department of Pathology, School of Medicine, Qinghai University, Xining 810001, China.; ^10^ School of Intelligent Medicine, Chengdu University of Traditional Chinese Medicine, Chengdu 611137, China.

## Abstract

The infiltration of myeloid-derived suppressor cells (MDSCs) is critical for the establishment of immunosuppressive tumor microenvironment (TME), yet no approved therapies specifically block it. Here, we employed a gene signature-based drug screening approach to identify potential agents for reversing MDSC-mediated immunosuppression in triple-negative breast cancer (TNBC). Transcriptomic analysis of 73,326 tumor samples and 190,588 single cells revealed C-X-C motif ligand 1 (*CXCL1*) and *CXCL2* as the key gene signature of MDSC infiltration. Combining this gene signature with high-throughput sequencing-based high-throughput screening (HTS^2^), we identified ponatinib as a potential inhibitor of MDSC infiltration. By employing multiple preclinical models, we demonstrated that ponatinib blocks MDSC infiltration and reverses the immunosuppressive TME, thus inhibiting TNBC growth in a TME-dependent manner, and significantly enhances anti-programmed cell death-ligand 1 (PD-L1) immunotherapy efficacy. Mechanistically, ponatinib directly inhibits p38α kinase activity, reducing signal transducer and activator of transcription 1 (STAT1) phosphorylation at Ser^727^ and suppressing *CXCL1* and *CXCL2* expression in cancer cells, thereby blocking MDSC infiltration. Our findings establish ponatinib as a novel inhibitor of MDSC-mediated immunosuppressive TME and underscore its therapeutic potential in combination with immune checkpoint blockade for TNBC treatment.

## Introduction

Cancer immunotherapies have revolutionized oncology by harnessing the immune system to combat tumor progression [1–3]. Among these approaches, immune checkpoint blockade (ICB) therapies, which targets regulatory molecules such as cytotoxic T lymphocyte-associated protein 4 (CTLA-4) and programmed cell death protein 1 or its ligand (PD-1/PD-L1), have demonstrated remarkable clinical efficacy across multiple cancer types [4–6]. However, only a small subset of patients responds to these immune checkpoint inhibitors (ICIs), leading to limited efficacy [7]. Emerging evidence suggests that the low objective response rate and poor survival outcomes observed in many solid tumors are closely linked to an immunosuppressive tumor microenvironment (TME) [8,9]. As such, combination immunotherapy strategies that target the immunosuppressive TME are critical to improving the efficacy and responsiveness of ICB therapies [10–13].

Tumors are frequently categorized into inflamed (immune-hot) or non-inflamed (immune-cold) phenotypes based on their immune infiltration profiles [14,15]. Non-inflamed tumors are typically marked by a highly immunosuppressive microenvironment [16], characterized by limited T cell presence and a predominance of immunosuppressive myeloid cells. Among these, myeloid-derived suppressor cells (MDSCs) and tumor-associated macrophages (TAMs) represent key contributors that actively inhibit T cell recruitment and cytotoxic function [17–20]. A growing body of evidence across diverse cancer types implicates intratumoral MDSCs as key mediators of immune resistance, facilitating tumor progression and metastasis [21–23]. Clinically, elevated MDSC levels in peripheral blood have been associated with higher tumor burden and reduced overall survival (OS) in multiple malignancies, including colorectal cancer [24], non-small cell lung cancer (NSCLC) [25], liver cancer [26,27], melanoma [28], thyroid cancer [29], and bladder cancer [30]. These findings underscore the potential of MDSCs as therapeutic targets in cancer immunotherapy. This is especially pertinent to TNBC, which lacks estrogen receptor (ER), progesterone receptor (PR), and human epidermal growth factor receptor 2 (HER2) expression and represents a prototypical immune-cold tumor type [31,32]. TNBC accounts for ~15% of breast cancers and is associated with higher rates of metastasis and mortality compared to hormone receptor-positive subtypes [33]. Its poor response to immunotherapies is largely attributed to a highly immunosuppressive microenvironment, underscoring a pressing clinical need for strategies targeting MDSCs and related pathways [34].

While strategies aimed at depleting MDSCs could enhance the efficacy of ICB, there are currently no approved agents that selectively eliminate MDSCs or prevent their infiltration into tumors, and standardized screening platforms for such drugs remain lacking.

MDSCs consist of 2 distinct subsets: polymorphonuclear MDSCs (PMN-MDSCs) and monocytic MDSCs (M-MDSCs) [35,36]**.** The development of these subsets is driven by selective cytokines and tumor-associated factors such as interleukin-6 (IL-6), IL-1β, tumor necrosis factor-α (TNF-α), granulocyte-macrophage colony-stimulating factor (GM-CSF), nuclear factor κB (NF-κB), and signal transducer and activator of transcription (STAT), among others [37,38]. Importantly, chemokines and their receptors, including the CXCL1/2–CXCR2 and CCL5–CCR5 axis, promote the recruitment of MDSCs to the TME [39,40]. Previous studies have shown that compounds induced by T helper 1 (Th1) chemokines can convert immunologically non-inflamed tumors into inflamed tumors, thereby enhancing the therapeutic response against cancer cells [41–43]. Therefore, combination therapies involving small molecules that inhibit MDSC infiltration into the TME represent a promising strategy for treating non-inflamed tumors.

Gene signatures of tissues or cells have broad applications in cancer research, including the identification of cancer metastasis [44] and classification of cancer subtypes [45]. These signatures have been used to predict clinical outcomes [46], assess tumor purity, estimate the abundance of immune cell types [47], and forecast responses to cancer immunotherapy [48]. Additionally, gene expression profiling of cellular perturbations has been extensively applied in the discovery of novel anticancer drugs [49].

In this context, we developed a gene signature specifically designed to represent high infiltration of MDSCs in tumors. We utilized the expression alterations of this gene signature as a screening metric in high-throughput pharmacological profiling. Our approach employs HTS^2^ [50] for the identification of therapeutic agents that synergize with immunotherapies. HTS^2^, which leverages next-generation sequencing technologies, has proven successful in uncovering compounds effective against prostate cancer [50], breast cancer [51–53], and liver cancer [54,55]. The goal of this study was to identify immunotherapy combination agents capable of inhibiting MDSC infiltration and enhancing immunotherapy efficacy in TNBC.

## Results

### Elevated *CXCL1* and *CXCL2* expression is linked to unfavorable clinical outcomes across diverse cancer types

**Fig. 1.**
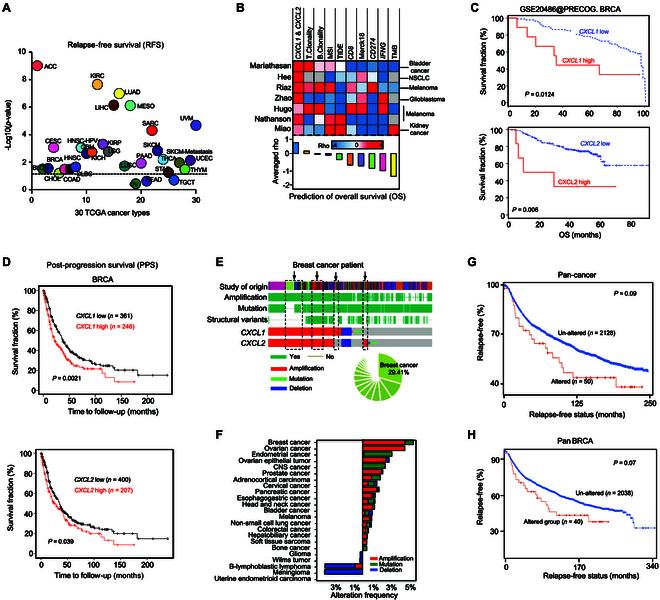
High *CXCL1* and *CXCL2* expression is associated with poor prognosis in patients across multiple cancers. (A) Impact of *CXCL1* and *CXCL2* expression on RFS in 2,178 patients across 30 cancer types. Log-rank *P* values were calculated to assess significance. The dashed line indicates *P* = 0.05. (B) Significance of 9 widely used ICB response biomarkers collected from TIDE [109] in relation to OS across 7 datasets from indicated studies. Red cells: Rho > 0, indicating increased risk. Rho values in the bottom panel represent the average for each biomarker across the 7 datasets (Table S1). *CXCL1* & *CXCL2*, average expression level of *CXCL1* and *CXCL2*; MSI, microsatellite instability signature; TIDE, Tumor Immune Dysfunction and Exclusion signature; Merck18, T cell-inflamed signature; TMB, tumor mutation burden. (C) KM plots of OS for patients with breast cancer, grouped by high or low expression of *CXCL1* (top) and *CXCL2* (bottom). *P* values were calculated using the log-rank test. Data were derived from RNA-seq and obtained from the KM plotter [113] and TIDE [109,111,112]. (D) KM plots of post-progression survival (PPS) for 607 breast cancer patients, grouped by high or low expression of *CXCL1* (top) and *CXCL2* (bottom). *P* values were calculated using the log-rank test. Best cutoffs were autocalculated and selected by the KM plotter tool [113]. (E) Genomic alterations of *CXCL1* and *CXCL2* among all TCGA patients across 30 cancer types. The pie chart shows the percentage of patients with breast cancer and other cancer types. (F) Distribution of the frequency of genomic alterations of *CXCL1* and *CXCL2* in TCGA cancer patients across 30 cancer types. (G and H) KM plots showing the impact of *CXCL1* and *CXCL2* genomic alterations on RFS in TCGA patients. Patients were grouped by the presence or absence of *CXCL1* and *CXCL2* genomic alterations across all TCGA cancer types (G; pan-cancer) or all breast cancer types (H; pan-BRCA). *P* values were determined using the log-rank test.

CXCL1 and CXCL2 are chemokines known to promote an immunosuppressive TME, as demonstrated in prior studies [56,57]. To investigate the prognostic relevance of these genes, we conducted a multi-level analysis using publicly available datasets from cancer patients. We first evaluated the association between *CXCL1*/*CXCL2* expression and patient survival outcomes across more than 30 cancer types from The Cancer Genome Atlas (TCGA) (Fig. 1A and B and Table S1). The expression levels of *CXCL1* and *CXCL2* were found to be predictive of both relapse-free survival (RFS) (Fig. 1A) and OS (Fig. 1B). Notably, patients with higher expression levels of *CXCL1* and *CXCL2* showed significantly poorer OS (Fig. 1B and C and Fig. S1A to E) and post-progression survival (PPS) (Fig. 1D), suggesting a consistent association between these chemokines and worse clinical outcomes across various cancer types. Additionally, we examined the genomic alterations of *CXCL1* and *CXCL2* in these cancers (Fig. 1E and F and Fig. S1F). Amplification of both genes was observed in several cancer types (Fig. 1E), with breast cancer showing the highest frequency of *CXCL1* and *CXCL2* amplifications (Fig. 1E and F). Genomic alterations (Fig. 1E and F) of the *CXCL1* and *CXCL2* gene signatures were linked to reduced RFS (Fig. 1G and H). These data indicated that higher expression of *CXCL1* and *CXCL2* is associated with poor prognosis across a range of cancer types.

### HTS^2^ screening identifies ponatinib as an antagonist of *CXCL1* and *CXCL2* expression in breast and colon cancer cells

**Fig. 2.**
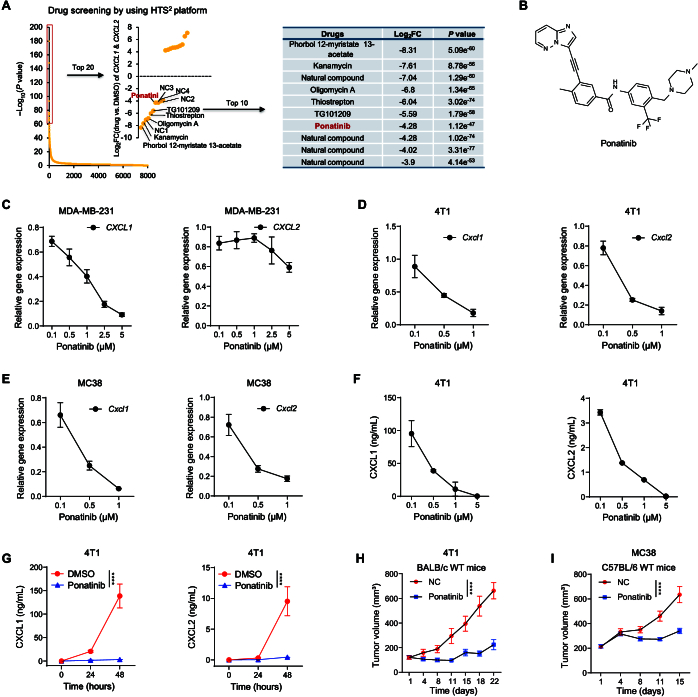
HTS^2^ screening identified ponatinib as an antagonist of *CXCL1* and *CXCL2* expression in diverse cancer cells. (A) Workflow of the drug screening for *CXCL1* and *CXCL2* using the HTS^2^ platform. The compounds were ranked based on their ability to down-regulate the expression of *CXCL1* and *CXCL2*. The significance of the difference between individual compounds and DMSO was calculated using Student’s *t* test. −Log_10_(*P* value) and log_2_(fold change) were used for compound ranking. Compounds below the dashed line represent the top 10 compounds that effectively decreased the expression of *CXCL1* and *CXCL2*. (B) Chemical structure of ponatinib (AP24534). (C and D) RT-qPCR analysis of MDA-MB-231 (C) and 4T1 (D) breast cancer cells treated with the indicated concentrations of ponatinib for 24 h. (E) RT-qPCR analysis of MC38 colon cancer cells treated with the indicated concentrations of ponatinib for 24 h. For (C) to (E), values were normalized to the vehicle (DMSO) control group values. Data are presented as means ± SD. (F and G) ELISA analysis of CXCL1 and CXCL2 protein levels in supernatant from 4T1 cells treated with the indicated concentrations of ponatinib (F) or with 1 μM ponatinib at the indicated time points (G). Data are presented as means ± SD. Statistical significance was determined by 2-way ANOVA. ****P < 0.0001. (H and I) Growth of 4T1 tumors in BALB/c WT mice (*n* = 6 mice per group) (H) or MC38 tumors in C57BL/6 WT mice (*n* = 10 mice per group) (I) treated with ponatinib or vehicle. Statistical significance was determined by 2-way ANOVA. ****P < 0.0001. Data are presented as means ± SEM of the indicated number of mice.

Given the positive correlation between high expression of *CXCL1* and *CXCL2* and poor patient prognosis across more than 30 cancer types, we thus considered the signature of *CXCL1* and *CXCL2* as an informative marker to identify potential agents capable of reversing the immunosuppressive TME. A high-throughput drug screening was conducted using the HTS^2^ platform [43,51,58], which screened a compound library [51] of 8,199 agents, including Food and Drug Administration (FDA)-approved drugs, epigenetic inhibitors, and natural products (Fig. 2A). Compounds were scored based on their ability to reduce *CXCL1* and *CXCL2* expression (Fig. 2A) [49,59]. Among the highest-ranked compounds were ponatinib, phorbol 12-myristate 13-acetate, kanamycin, oligomycin A, thiostrepton, TG101209, and 4 natural compounds (Fig. 2A). Notably, several of these compounds, including kanamycin, oligomycin A, thiostrepton, and phorbol 12-myristate 13-acetate, have been previously linked to the regulation of chemokine production [60–62]. This result demonstrates that our approach successfully identifies compounds that modulate *CXCL1* and *CXCL2* expression.

Based on our previous findings that ponatinib (Fig. 2B), an FDA-approved multi-targeted tyrosine kinase inhibitor for leukemia [63], inhibits breast cancer lung metastasis [51], we selected ponatinib for further investigation. Using reverse transcription quantitative polymerase chain reaction (RT-qPCR), we assessed ponatinib-induced changes in *CXCL1* and *CXCL2* expression in multiple cancer cell lines (5 breast cancer and 2 colorectal cancer cell lines). Ponatinib consistently down-regulated the expression of *CXCL1* and *CXCL2* across all cell lines examined (Fig. 2C to E and Fig. S2A). Additionally, enzyme-linked immunosorbent assay (ELISA) of 4T1 cell exudates confirmed that ponatinib treatment significantly reduced CXCL1 and CXCL2 protein levels (Fig. 2F and G).

We next assessed the antitumor effects of ponatinib in murine models of breast cancer (4T1) and colorectal cancer (MC38). Briefly, 4T1 cells were orthotopically implanted into the mammary fat pads of BALB/c wild-type (WT) mice, while MC38 cells were inoculated subcutaneously into the right flank of C57BL/6 WT mice. Tumor-bearing mice received either ponatinib or a vehicle control. After 3 weeks of treatment in the 4T1 model (Fig. S2B), both tumor volume (Fig. 2H) and weight (Fig. S2C) were significantly reduced in the ponatinib group, with inhibition ratios of 66.2% and 73.3%, respectively (*P* < 0.001). Similar results were observed in the MC38 model following 2 weeks of treatment (Fig. S2D), where ponatinib markedly suppressed tumor growth, as evidenced by decreased tumor volume and weight (Fig. 2I and Fig. S2E). These results confirm that ponatinib, identified through HTS^2^ screening as an antagonist of CXCL1 and CXCL2 expression, exerts potent antitumor effects in both breast and colorectal cancer models.

### Ponatinib inhibits *CXCL1* and *CXCL2* expression by disrupting p38α-induced STAT1 phosphorylation

We next investigated whether ponatinib inhibits *CXCL1* and *CXCL2* expression through the previously reported targets SRC and/or ABL [63]. However, no changes in *CXCL1* or *CXCL2* expression were observed following short hairpin RNA (shRNA)-mediated knockdown of *SRC*, *ABL1*, or *ABL2* in cancer cells (Fig. S3A and B), suggesting that the inhibitory effects of ponatinib on *CXCL1* and *CXCL2* are mediated by alternative targets or pathways.

**Fig. 3.**
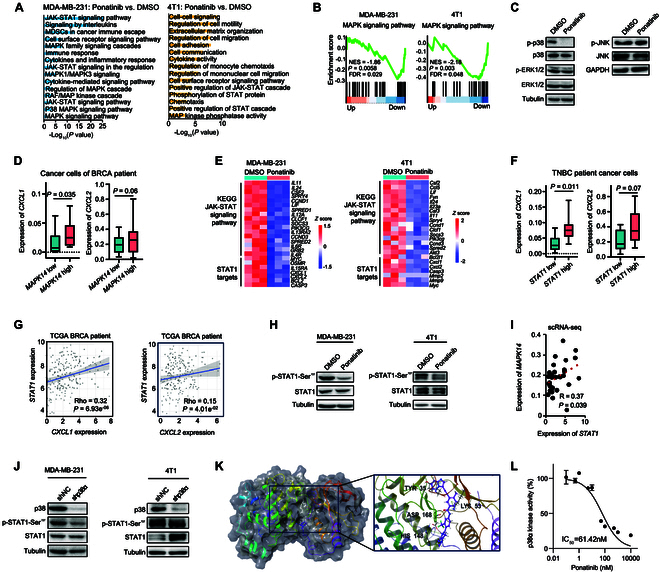
Ponatinib inhibits *CXCL1* and *CXCL2* expression through the p38α–STAT1 signaling pathway. (A) GO analysis of DEGs between ponatinib-treated versus DMSO-treated MDA-MB-231 (left) and 4T1 (right) cells. DEGs were defined as genes with log_2_(fold change) < −1 and *P* < 0.05, based on RNA-seq data. *P* values were determined employing the Fisher’s exact test. (B) Enrichment analysis of the MAPK signaling pathway in MDA-MB-231 (left) and 4T1 (right) cells treated with ponatinib versus DMSO. Data were derived from RNA-seq analysis. NES, normalized enrichment score; *P*, nominal *P* value; FDR, false discovery rate. *P* values were determined using the Kolmogorov–Smirnov (KS) test. (C) Immunoblotting of the indicated proteins in MDA-MB-231 cells treated with 1 μM ponatinib or DMSO. (D) Box plots showing the expression levels of *CXCL1* and *CXCL2* in cancer cells from breast cancer patients, comparing groups with low expression of *MAPK14* to groups with high expression (normalized counts > 0). Data from publicly available scRNA-seq data from 29 breast cancer patients [65]. *P* values were determined using the Mann–Whitney test. (E) Heatmap of genes involved in the JAK–STAT signaling pathway (top) and STAT1 target genes (bottom) down-regulated by ponatinib in MDA-MB-231 and 4T1 cell lines. (F) Box plots showing the expression levels of *CXCL1* or *CXCL2* in cancer cells from breast cancer patients, comparing groups with low versus high expression of *STAT1* (normalized counts > 0). *P* values were determined using the Mann–Whitney test. (G) Correlation of *CXCL1* or *CXCL2* expression with *STAT1* expression in TCGA breast cancer patients. Each dot represents an individual patient. Rhos were calculated using TIMER version 2 [72]. *P* values were determined using the Spearman correlation test. (H) Immunoblotting of the indicated proteins in lysates from MDA-MB-231 cells (left) or 4T1 cells (right) treated with 1 μM ponatinib or vehicle (DMSO). (I) Correlation of the expression levels of *MAPK14* (encoding p38α) with *STAT1* expression in tumor cells. Each dot represents a breast cancer patient, with the average expression level of all cancer cells from that patient used as the expression level for that individual [65]. *P* values were determined using the Pearson’s correlation test. (J) Immunoblotting of the indicated proteins in MDA-MB-231 (left) or 4T1 (right) cells with p38α knockdown compared to negative control. (K) Three-dimensional interaction diagram of ponatinib docked in p38α MAPK kinase domain (active site) generated by Schrödinger Maestro, highlighting key binding interactions. (L) In vitro activity of p38α exposed to the indicated concentrations of ponatinib. Data were fitted using a nonlinear regression model (GraphPad Prism) to determine the IC_50_ values.

To explore this further, we performed RNA sequencing (RNA-seq) to examine the transcriptome-wide effects of ponatinib in both MDA-MB-231 and 4T1 cells. Gene Ontology (GO) analysis of down-regulated genes revealed significant enrichment of the mitogen-activated protein kinase (MAPK) signaling pathway in ponatinib-treated cells compared to controls (Fig. 3A). Gene Set Enrichment Analysis (GSEA) also highlighted MAPK signaling as enriched in the ponatinib-treated groups (Fig. 3B). Immunoblotting to assess the phosphorylation of key MAPK components, including extracellular receptor tyrosine kinase (ERK), p38, and c-Jun N-terminal kinase (JNK) [64], in MDA-MB-231 cells showed a marked decrease in phosphorylated p38 levels following ponatinib treatment (Fig. 3C and Fig. S4A to E). Additionally, single-cell RNA sequencing (scRNA-seq) analysis of publicly available breast cancer datasets revealed that high expression of *MAPK14* (p38α), a member of p38 family, is positively correlated with elevated *CXCL1* and *CXCL2* transcription in cancer cells from 29 breast cancer patients [65] (Fig. 3D). Furthermore, the expression of *CXCL1* and *CXCL2* was also inhibited by 2 other p38 inhibitors (Fig. S5A).

Kyoto Encyclopedia of Genes and Genomes (KEGG) pathway analysis of RNA-seq data revealed significant enrichment of the Janus kinase (JAK)–STAT signaling pathway in ponatinib-treated cells compared to the controls (Fig. 3A), with a concurrent down-regulation of genes involved in this pathway (Fig. 3E). Notably, the promoter regions of both *CXCL1* and *CXCL2* contain multiple binding sites for STAT1, as reported in a previous study [66]. Our data showed that knocking down *STAT1* significantly decreased the mRNA levels of *CXCL1*, with a similar trend observed for *CXCL2* (Fig. S5B and C). Furthermore, the expression of STAT1 target genes, including *CXCL1* and *CXCL2* [66], was also reduced in ponatinib-treated breast cancer cells compared to the controls (Fig. 3E). Additionally, high *STAT1* expression was positively correlated with increased *CXCL1* and *CXCL2* levels in cancer cells from TNBC patients (Fig. 3F), as well as in breast cancer (BRCA) patients from TCGA (Fig. 3G) and Clinical Proteomic Tumor Analysis Consortium (CPTAC) (Fig. S5D) cohorts [67]. Notably, spatial transcriptomics (ST) data revealed that *CXCL1* and *CXCL2* were colocalized with *STAT1* in tumor cell-enriched regions from a BRCA patient [68] (Fig. S5E). We also observed that ponatinib treatment markedly decreased STAT1 phosphorylation at Ser^727^ in MDA-MB-231 cells and 4T1 breast cancer cells (Fig. 3H), as well as in multiple other cancer cell lines (Fig. S4C and D).

We further explored the relationship between p38 and STAT1 in breast cancer. scRNA-seq analysis showed that high *STAT1* expression was associated with elevated *MAPK14* expression in cancer cells from TNBC patients (Fig. 3I). A previous study has shown that phosphorylation of STAT1 at Ser^727^ is positively associated with the p38 MAPK signaling pathway [69]. Consistent with this, our immunoblotting analysis demonstrated that knocking down p38 markedly reduced STAT1 phosphorylation at Ser^727^ (Fig. 3J). Notably, the knockdown of *SRC* and *ABL* in MDA-MB-231 and 4T1 cells showed no appreciable modulation of phospho-STAT1 levels, mechanistically distinguishing the specific regulatory role of p38 in STAT1 activation from these established tyrosine kinase targets of ponatinib (Fig. S5F and G).

Taken together, these results suggest that ponatinib inhibits *CXCL1* and *CXCL2* expression through the p38–STAT1 signaling pathway.

### Ponatinib inhibits the kinase activity of p38α

Given that ponatinib reduced the phosphorylation of p38 in various cancer cell lines, we next investigated whether ponatinib directly binds to p38 and affects its kinase activity. Molecular docking analysis revealed a high binding affinity between ponatinib and p38α, with the lowest docking score reflecting the free energy of binding (Fig. 3K and Table S2). To confirm this, we performed a p38α kinase assay, which demonstrated that ponatinib inhibits p38α kinase activity in a dose-dependent manner (Fig. 3L). Together, these findings suggest that ponatinib might directly bind to p38α, inhibiting its phosphorylation and kinase activity. This, in turn, reduces STAT1 phosphorylation and the subsequent expression of *CXCL1* and *CXCL2*.

### Ponatinib inhibits TNBC growth in immunocompetent and nude mice but not in NSG mice

**Fig. 4.**
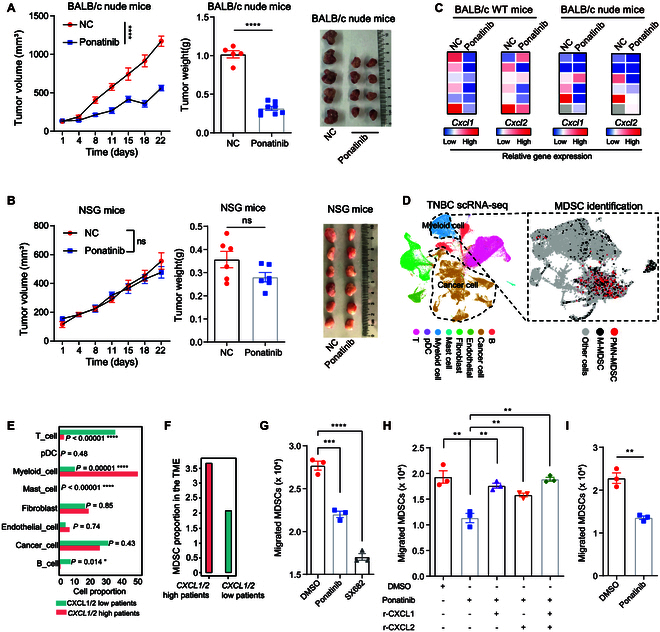
Ponatinib inhibits the infiltration of immunosuppressive MDSCs into TNBC TME by repressing *CXCL1* and *CXCL2* expression. (A) Growth of 4T1 tumors in BALB/c nude mice treated with ponatinib or vehicle (control, *n* = 5; ponatinib, *n* = 8). Tumor volume kinetics were monitored by vernier calipers (left). Statistical significance was determined by 2-way ANOVA. Terminal tumor weight quantification is shown (middle), with statistical significance determined by unpaired 2-tailed Student’s *t* tests. ****P < 0.0001. The tumor image (right) shows 4T1 tumors from the indicated groups. (B) Growth of 4T1 tumors in NSG mice treated with ponatinib or vehicle (*n* = 6 mice per group). Tumor volume kinetics monitored by vernier calipers (left). Statistical significance was determined by 2-way ANOVA. Terminal tumor weight quantification is shown (middle), with statistical significance determined by unpaired 2-tailed Student’s *t* tests. ns (not significant), *P* > 0.05. The tumor image (right) shows 4T1 tumors from the indicated groups. (C) RT-qPCR analysis of *Cxcl1* and *Cxcl2* mRNA levels in 4T1 tumors from BALB/c WT mice (left) or nude mice (right) receiving ponatinib or control treatments as described in Figs. 2H and 4A. Heatmap representing the relative expression of the indicated chemokine genes normalized to *Gapdh*. Expression scaled from high (red) to low (blue). Each square represents individual tumors from a single mouse (gray squares indicate tumors from mice sacrificed due to the requirement of animal ethics). (D) MDSC identification using publicly available scRNA-seq data from breast cancer patients. Cells were colored by cell types defined in a breast cancer scRNA-seq dataset [65] (left) and by MDSC types predicted using the scPred package [70] (right). MDSCs were identified in a dataset of 29 breast cancer patients receiving ICB therapy (EGAD00001006608) [65] by training on another well-defined breast cancer MDSC dataset (GSE139125) [71]. (E) Percentage of different cell types in the breast cancer TME, grouped by high or low expression of *CXCL1* and *CXCL2* in scRNA-seq data from breast cancer patients [65]. *P* values were determined using the chi-square test. (F) Percentage of MDSCs (relative to all myeloid cells) in breast cancer patients with high or low expression of *CXCL1* and *CXCL2*, calculated using the breast cancer scRNA-seq dataset [65]. (G) Migration of MDSCs toward CM from 4T1 cells treated with ponatinib or DMSO, evaluated using in vitro Transwell migration assays. (H) Migration of MDSCs toward CM from 4T1 cells treated with either DMSO or ponatinib and supplemented with or without recombinant mouse CXCL1 and CXCL2. (I) Transwell migration assays for MDSC migration toward CM from MC38 cells treated with either ponatinib or DMSO. Data are presented as means ± SEM. Statistical significance was assessed using unpaired 2-tailed Student’s *t* tests. **P < 0.01, ***P < 0.001, and ****P < 0.0001.

We observed significant tumor inhibition with ponatinib treatment in both breast cancer and colon cancer models in WT mice (Fig. 2H and I). To determine whether the antitumor effects of ponatinib are mediated by immune cells in the TME, we utilized 2 immune-deficient mammary tumor models. The antitumor efficacy of ponatinib was assessed in 4T1 tumors transplanted into BALB/c nude mice (which lack T cells) and NOD scid gamma (NSG) mice [which lack T cells, natural killer (NK) cells, and B cells]. Upon intramammary injection, 4T1 tumors in both BALB/c nude mice and NSG mice colonized to a similar extent as those in WT mice. After ponatinib treatment, tumor volume and weight were significantly suppressed in nude mice (52.0% and 69.0% inhibition, respectively; *P* < 0.0001) (Fig. 4A). In contrast, ponatinib treatment in NSG mice did not result in significant changes in tumor volume and weight (Fig. 4B). Comparative analysis of syngeneic tumor models reveals that while ponatinib significantly suppresses 4T1 tumor growth in both WT and T cell-deficient nude mice, its antitumor efficacy is attenuated in the immunocompromised cohort (tumor volume: 66.2% versus 52.0%; tumor weight: 73.3% versus 69.0%) (Figs. 2H and 4A). This intermodel divergence (Δ14.2% tumor volume inhibition) implicates a nonessential but modulatory role for T cell-mediated adaptive immunity within the 4T1 TME. Strikingly, the therapeutic response to ponatinib was preserved in lymphocyte-impaired nude mice, in sharp contrast to its complete abrogation in pan-lymphopenic NSG models. This contrast mechanistically demonstrates that the antitumor activity of ponatinib operates through adaptive T cell-independent pathways while requiring residual innate immune components. Collectively, these findings indicate that the anticancer effects of ponatinib are dependent on the immune cells within the TME.

### TNBC inhibition of ponatinib depends on MDSC infiltration by suppressing the expression of *CXCL1* and *CXCL2* in TNBC cells

RT-qPCR analysis showed that ponatinib inhibits the expression of *Cxcl1* and *Cxcl2* in 4T1 tumors (Fig. 4C), which is consistent with our in vitro results (Fig. 2C to E and Fig. S2A). Given that *CXCL1* and *CXCL2* act as important mediators of chemotaxis [56,57], including the recruitment of MDSCs, we next analyzed the relationship between *CXCL1* and *CXCL2* and MDSC infiltration using scRNA-seq data. We applied scPred, a supervised machine learning-based method for cell type prediction [70], to train a classifier using a breast cancer scRNA-seq dataset with well-defined MDSC populations [71] (Fig. 4D and Fig. S6A to C).

We subsequently applied the classifier to an independent cohort of breast cancer patients [65] (Fig. 4E and Fig. S6A to C). Our analysis revealed a strong correlation between elevated *CXCL1* and *CXCL2* expression and increased proportions of myeloid cells in the TME (Fig. 4E). Notably, the percentage of MDSCs was positively associated with *CXCL1* and *CXCL2* expression levels (Fig. 4F). This finding was further supported by bulk RNA-seq analysis using the TIMER tool [72] (Fig. S6D). Additionally, *CD33*, a commonly used marker for identifying human MDSCs [73], showed a positive correlation with *CXCL1*/*CXCL2* expression in both TCGA [72] (Fig. S6E) and CPTAC BRCA patients [67] (Fig. S6F). Immunohistochemistry (IHC) analysis of 129 TNBC samples showed that patients with elevated CXCL2 expression had significantly higher CD33 protein levels (Table S3 and Fig. S6G and H).

Furthermore, *CXCL1* and *CXCL2* expression was positively linked to 15 immunosuppressive molecules [74,75] known to mediate MDSC function in breast cancer (Fig. S7A to D), as well as across 40 cancer types in TCGA (Fig. S7E and F). Additionally, a low percentage of T cells (Fig. S7G and H) and decreased expression of T cell functional genes (Fig. S7I) were observed in *CXCL1* and *CXCL2* highly expressed patients. This is potentially due to the recruitment inhibition caused by *CXCL1*- and *CXCL2*-mediated MDSC infiltration (Fig. 4E and F).

These findings indicate that *CXCL1* and *CXCL2* play a crucial role in MDSC infiltration and its immunosuppressive function, highlighting their essential contribution to the immunosuppressive TME.

To assess the impact of *CXCL1* and *CXCL2* on MDSC recruitment under ponatinib treatment, we performed a migration assay using freshly isolated MDSCs from 4T1 tumor-bearing mice. MDSCs were cultured in the upper chamber of transwells, and 4T1 conditioned medium (CM) was added to the lower chamber to analyze the effect of ponatinib on MDSC migration. As expected, ponatinib treatment of 4T1 cells significantly reduced MDSC migration toward the growth medium (Fig. 4G). We also evaluated the role of the CXCR2 receptor, which is crucial for MDSC trafficking into the TME [76]. The addition of the CXCR2 inhibitor SX-682 [77,78] to the upper chamber significantly reduced MDSC migration (Fig. 4G). Furthermore, the involvement of CXCL1 and CXCL2 in MDSC recruitment under ponatinib treatment was confirmed by observing increased MDSC migration upon the addition of recombinant mouse CXCL1 or CXCL2 protein to the growth medium of ponatinib-treated 4T1 cells (Fig. 4H). Similar results were observed in colon cancer models, where ponatinib treatment of MC38 cells also significantly reduced MDSC migration in vitro (Fig. 4I).

Together, these findings suggest that ponatinib-mediated inhibition of *CXCL1* and *CXCL2* expression in cancer cells reduces MDSC infiltration into the TME, thereby diminishing the immunosuppressive effects and supporting its potential as an immunotherapy enhancer.

### Ponatinib-induced TNBC inhibition relies on MDSC infiltration in the TME

**Fig. 5.**
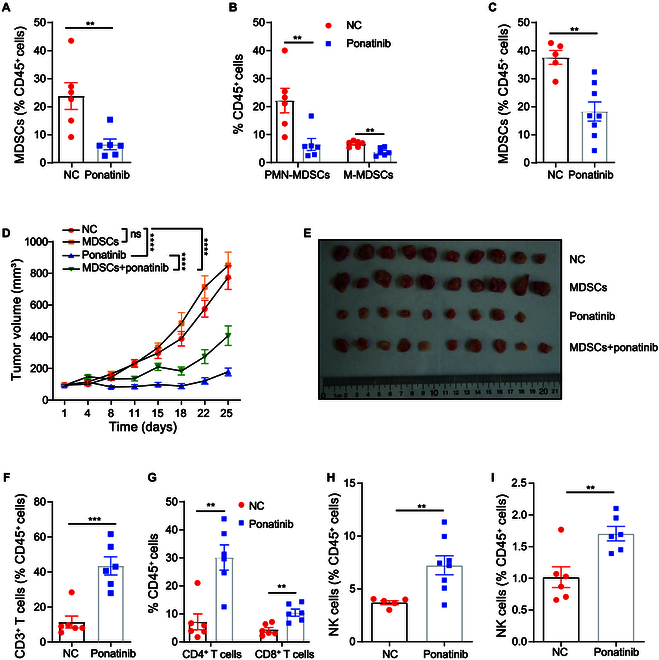
Ponatinib reduces the infiltration of protumor-related immune cells and enhances the infiltration of antitumor-related immune cells into the TME in vivo. (A) Flow cytometry analysis of MDSCs (CD45^+^CD11b^+^F4/80^−^Gr-1^+^) in 4T1 tumors of BALB/c WT mice treated with ponatinib or vehicle. (B) Flow cytometry analysis of PMN-MDSCs (CD45^+^CD11b^+^Ly-6G^+^Ly-6C^−^) and M-MDSCs (CD45^+^CD11b^+^Ly-6G^−^Ly-6C^+^) in 4T1 tumors of BALB/c WT mice treated with ponatinib or vehicle. (C) Flow cytometry analysis of MDSC in 4T1 tumors from BALB/c nude mice treated with ponatinib or vehicle. Data are presented as means ± SEM. Statistical significance was determined using unpaired 2-tailed Student’s *t* tests. **P < 0.01. (D) A mixture of 4T1 cells and MDSCs (isolated from tumor-bearing mice) was inoculated into the fat pad of BALB/c WT mice (*n* = 10). The growth of the tumor was monitored, and ponatinib (30 mg/kg) or vehicle was administered 4 days a week when the tumor volume reached 100 mm^3^. Data represent mean ± SEM. Two-way ANOVA was used for statistical significance. ns, *P* > 0.05, ****P < 0.0001. (E) Representative images of 4T1 tumors from the indicated groups in (D). (F) Flow cytometry analysis of CD3^+^ T cells (CD45^+^CD3e^+^) in 4T1 tumors of BALB/c WT mice treated with ponatinib or vehicle. (G) Flow cytometry analysis of CD4^+^ T cells (CD45^+^CD3e^+^CD8^−^CD4^+^) and CD8^+^ T cells (CD45^+^CD3e^+^CD8^+^CD4^−^) in 4T1 tumors of BALB/c WT mice treated with ponatinib or vehicle. (H) Flow cytometry analysis of NK cells (CD45^+^CD49b^+^) in 4T1 tumors from BALB/c nude mice treated with ponatinib or vehicle. (I) Flow cytometry data analysis of NK cells (CD45^+^Nkp46^+^) in spleens from 4T1 tumor-bearing BALB/c WT mice treated with ponatinib or vehicle. Data are presented as means ± SEM. Statistical significance was determined using unpaired 2-tailed Student’s *t* tests. **P < 0.01, ***P < 0.001.

To evaluate the TME-related antitumor effects of ponatinib, we investigated changes in the TME following ponatinib treatment. Flow cytometry was employed to assess the distribution and trafficking of tumor-infiltrating leukocytes in immunecompetent mouse models. Our results revealed a significant reduction in the proportion of MDSCs within 4T1 tumors upon ponatinib treatment (Fig. 5A). This indicates that the antitumor activity of ponatinib is closely linked to its ability to alter MDSC infiltration in the TME.

We next investigated the subtypes of MDSCs infiltrated in 4T1 tumors of WT mice after ponatinib treatment. MDSCs are classified into PMN-MDSCs and M-MDSCs [36]. Our results revealed that ponatinib treatment led to a significant reduction in both PMN-MDSCs and M-MDSCs within the tumors (Fig. 5B). Additionally, in contrast to WT mice, a dramatic reduction in MDSC infiltration was also observed in 4T1 tumors from BALB/c nude mice (Fig. 5C). These results indicate that ponatinib inhibits tumor progression by reducing immune suppression within the TME. To determine whether the decreased MDSC presence in the TME was due to reduced infiltration or suppression of MDSC expansion in the immune system, we analyzed the MDSC population in the spleens of treated mice. Notably, there were no significant differences in the proportion of MDSCs in the spleens of either WT (Fig. S8A and B) or BALB/c nude mice (Fig. S8C) upon ponatinib treatment compared with the controls. These results indicate that the effect of ponatinib on MDSC reduction in the TME is primarily due to impaired infiltration rather than inhibition of MDSC expansion systemically.

To confirm that the antitumor effects of ponatinib are mediated through reduction of immune suppression by MDSCs, we performed an MDSC rescue experiment. MDSCs isolated from tumor-bearing mice were mixed with 4T1 cells and injected into the mammary fat pads of BALB/c mice. Our results showed that the presence of higher levels of intratumoral MDSCs did not significantly promote tumor growth in the absence of ponatinib treatment (Fig. 5D and E). However, elevated intratumoral MDSCs significantly diminish the antitumor effects of ponatinib (Fig. 5D and E). This suggests that the antitumor effect of ponatinib is dependent on the reduction of MDSC infiltration. Taken together, our findings indicate that the reduction of immunosuppressive MDSCs in the TME is essential for the antitumor effect of ponatinib.

### Ponatinib enhances the infiltration of antitumor immune cells into the TME in vivo

In addition to reducing MDSC infiltration, we also assessed the effect of ponatinib on antitumor immune cells in the TME. In WT mice, ponatinib significantly increased the infiltration of CD3^+^ T cells into 4T1 tumor sites compared to the controls (Fig. 5F and Fig. S8D). Both CD4^+^ and CD8^+^ T cells were notably enriched in the ponatinib-treated tumors (Fig. 5G), implying that ponatinib promotes a robust antitumor immune response in mammary tumors.

To further investigate the impact of ponatinib on immune cell populations, we examined 4T1 tumors in BALB/c nude mice, which lack T cells. Interestingly, despite the absence of T cells, ponatinib treatment led to a significant increase in the proportion of NK cells in the tumors (Fig. 5H). These results indicate that ponatinib can induce NK cell enrichment in the TME independently of T cells, further supporting its role in enhancing the antitumor immune response.

In addition to examining immune cell populations in tumors, we also evaluated the effect of ponatinib on immune cells in the spleens of tumor-bearing mice. Notably, ponatinib treatment led to a significant increase in NK cells in the spleens of 4T1 tumor-bearing WT mice compared to the controls (Fig. 5I). However, no significant differences were observed in the populations of CD3^+^ T cells, CD4^+^ T cells, or CD8^+^ T cells (Fig. S8E and F). Similarly, ponatinib treatment caused a marked increase in NK cells in the spleens of 4T1 tumor-bearing BALB/c nude mice (Fig. S8G), consistent with our findings in WT mice.

Collectively, these results suggest that ponatinib treatment leads to a notable increase in the infiltration of antitumor T cells and NK cells in the TME, enhancing the immune response within the mammary TME. This is consistent with our earlier observation that ponatinib inhibits MDSC infiltration into the TME. As MDSCs are known to suppress the proliferation and function of both T cells and NK cells [19,20,77], ponatinib’s reduction of MDSCs and its subsequent enhancement of antitumor immune cells likely contribute to the reprogramming of the TME from a protumor to an antitumor state. Importantly, ponatinib did not substantially alter immune cell populations in the spleens of mice, indicating that the immunomodulatory effects are specific to the TME rather than systemic immunity.

### Ponatinib enhances the efficacy of ICB therapy against TNBC in vivo

**Fig. 6.**
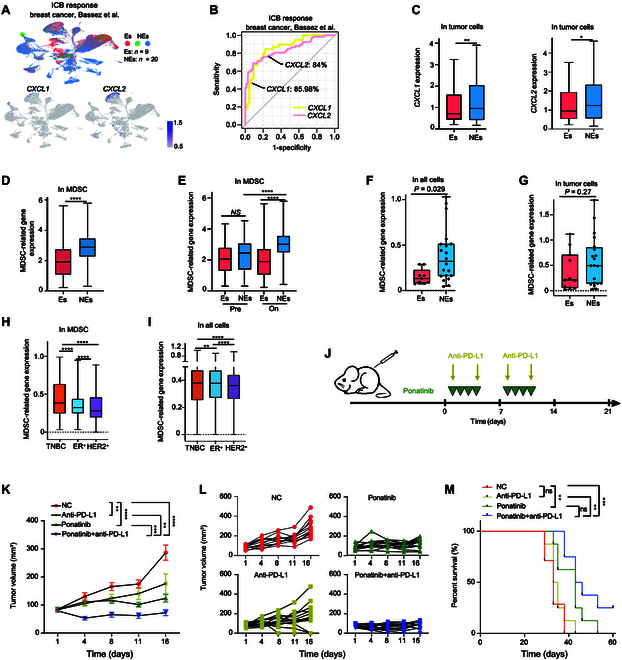
Combination treatment with ponatinib enhances the therapeutic efficacy of anti-PD-L1 against TNBC. (A) Expression levels of *CXCL1* or *CXCL2* and therapeutic responses assessed from publicly available scRNA-seq data for 29 breast cancer patients who received ICB therapy [65]. Cells were colored based on ICB response results [65] (top) and gene expression levels of *CXCL1* and *CXCL2* (bottom). Es, patients with clonotype expansion (*n* = 9); NEs, patients with limited or no clonotype expansion (*n* = 20). (B) Receiver operating characteristic (ROC) curves depicting the predictive value of *CXCL1* and *CXCL2* expression levels for anti-PD-1 therapy response in breast cancer patients. (C) *CXCL1* and *CXCL2* expression in tumor cells, comparing Es with NEs, calculated using the breast cancer scRNA-seq dataset [65]. *P* values were determined using the Mann–Whitney test. (D) Expression of known MDSC-related genes [71,74,75,79,80] in MDSCs determined in this study, comparing Es with NEs. MDSCs were identified in the breast cancer scRNA-seq dataset (EGAD00001006608) [65], through learning from another breast cancer scRNA-seq dataset with MDSC well-defined (GSE139125) [71], using the cell type prediction method of scPred package [70]. *P* values were determined using the Mann–Whitney test. (E) Expression of known MDSC-related genes [71,74,75,79,80] in MDSCs, comparing pretreatment with on-treatment conditions. Pre, biopsy collected before anti-PD-1 treatment; On, biopsy collected during subsequent surgery. *P* values were determined using the Mann–Whitney test. (F) Expression of known MDSC-related genes in the breast TME, comparing Es with NEs from the breast cancer scRNA-seq dataset [65]. *P* values were determined using the Mann–Whitney test. (G) Expression of known MDSC-related genes in tumor cells, comparing Es with NEs, calculated using the breast cancer scRNA-seq dataset [65]. *P* values were determined using the Mann–Whitney test. (H) Expression of known MDSC-related genes in MDSCs, comparing across 3 different breast cancer types. *P* values were determined using the Mann–Whitney test. (I) Expression of known MDSC-related genes in the TME, comparing across 3 different breast cancer types, was calculated using the breast cancer scRNA-seq dataset [65]. *P* values were determined using the Mann–Whitney test. (J) Schematic diagram of the combination treatment protocol for 4T1-bearing mice; ponatinib (30 mg/kg) or vehicle was administered 4 days per week, and PD-L1 monoclonal antibody (200 μg per injection, twice a week), either alone or in combination, starting when the tumor volume reached 100 mm^3^ (*n* = 12 to 14 mice per group). (K and L) Growth of 4T1 tumors in BALB/c WT mice treated with the indicated conditions from (J). Tumor volume kinetics were monitored by vernier calipers. Data represent mean ± SEM of *n* mice. Two-way ANOVA determined statistical significance. **P < 0.01, ***P < 0.001, and ****P < 0.0001. (M) Survival curves for the indicated groups. Statistical significance was determined using the log-rank (Mantel–Cox) test. ns, *P* > 0.05, **P < 0.01, and ***P < 0.001.

Given that ponatinib treatment inhibits MDSC infiltration into the TME and enhances T lymphocyte responses, potentially reprogramming the microenvironment to an antitumor state, we hypothesized that it could further improve the efficacy of ICB therapy. To test this, we analyzed the correlation between *CXCL1* and *CXCL2* expression and patient responses to ICB therapy using scRNA-seq data from breast cancer patients [65] (Fig. 6A). As anticipated, elevated expression of *CXCL1* and *CXCL2* showed strong predictive capacity for therapeutic response (Fig. 6B). *CXCL1* and *CXCL2* levels were significantly higher in tumor cells or all the cells in the TME of nonresponder patients with limited or no clonotype expansion (NEs), compared to patients with clonotype expansion (Es) (Fig. 6C and Fig. S9A and B). Further analysis revealed that the expression of *CXCL1* and *CXCL2* was consistently higher in nonresponders compared to responders (Fig. S9C). Additionally, we compared the predictive performance of the *CXCL1* and *CXCL2* signature with other published biomarkers for ICB response (Fig. S9D and E). Our results showed that the *CXCL1* and *CXCL2* signature outperformed 9 other ICB prediction methods across 8 different datasets (Fig. S9D and E).

In comparison to the Es group, MDSC-related genes [71,79,80], including those involved in immune suppression and MDSC functionality regulation, were expressed at relatively higher expression levels in the MDSC of NEs group (Fig. 6D and Fig. S9F). Further analysis of pretreatment and on-treatment groups showed that MDSC of NEs exhibited higher expression of MDSC-related genes [71,79,80] after receiving ICB treatments (Fig. 6E). This may partly explain the resistance to cancer immunotherapy observed in these patients. Consistent with the expression differences in MDSC, all cells in the TME and tumor cells of NEs displayed higher levels of MDSC-related genes (Fig. 6F and G).

Next, we examined MDSC-related gene [71,79,80] expression across 3 subtypes of breast cancer (Fig. 6H and I). We found that MDSC-related genes exhibited higher expression levels in MDSC and all cells in the TME of TNBC compared to HER2^+^ or ER^+^ breast cancer (Fig. 6H and I). Given the refractory nature of TNBC, these elevated levels of MDSC-related genes may contribute to the formation of a protumor TME. Thus, targeting *CXCL1*, *CXCL2*, or MDSCs may serve as a promising immune therapy strategy to reverse the protumor TME and shift it toward an antitumor environment.

To further validate this hypothesis, we conducted in vivo studies using a syngeneic tumor model in which 4T1 tumor-bearing mice were treated with ponatinib alone or in combination with an anti-PD-L1 antibody (Fig. 6J). Ponatinib or anti-PD-L1 monotherapy both suppressed tumor growth significantly (56.7% and 38.5% inhibition, respectively). However, combining ponatinib with anti-PD-L1 resulted in more pronounced tumor inhibition (74.6% inhibition) than monotherapy (Fig. 6K and L). Survival curve analysis confirmed that ponatinib significantly enhanced survival in 4T1 tumor-bearing mice receiving anti-PD-L1 treatment (Fig. 6M). These results demonstrate that ponatinib treatment sensitizes TNBC to checkpoint inhibitor-based immunotherapy by reshaping the TME from a non-inflamed to an inflamed state, thereby enhancing the antitumor immune response.

## Discussion

**Fig. 7.**
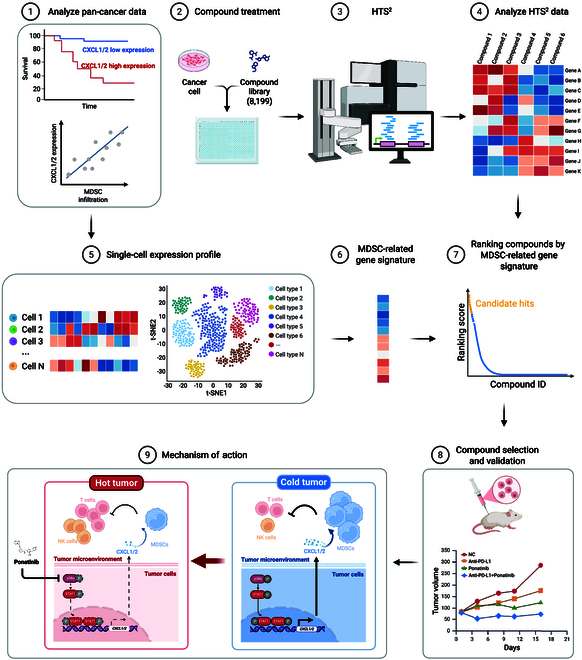
Ponatinib enhances immunotherapy in TNBC by reversing MDSC-driven immunosuppression identified via gene signature-based high-throughput screening (created in BioRender).

In this study, we identified ponatinib as a potential synergetic agent that enhances ICB therapy by leveraging a gene signature-based high-throughput screening approach. Specifically, our proof-of-concept experiments demonstrate that ponatinib improves antitumor immune response and potentiates ICB efficacy in TNBC by inhibiting the recruitment of MDSCs by targeting the p38–STAT1 signaling pathway (Fig. 7).

MDSC-driven immunosuppression represents a key obstacle in effective antitumor immunity and is widely recognized as a critical mechanism of tumor immune escape. Moreover, MDSCs are considered as a prominent contributor to resistance against therapies, including anti-PD-1/PD-L1 immunotherapy [81]. Despite the critical role MDSCs play in immune suppression, no high-throughput screening model currently exists to identify drugs that can effectively block MDSC infiltration and their immunosuppressive functions within tumors.

We introduced a gene signature-based high-throughput screening strategy for identifying potential compounds that target MDSC-mediated immunosuppressive microenvironments (Fig. 7), directly linking drug action to reprogramming the TME rather than simple phenotypic readouts. Firstly, we applied the scPred machine learning algorithm [70] to define MDSC [71] in a breast cancer scRNA-seq dataset. Subsequent analysis revealed key genes associated with both MDSC infiltration and immunosuppressive functionality as the MDSC-related gene signature. This gene signature, derived from tumors with high MDSC infiltration, enhances the identification of compounds capable of normalizing pathological TME conditions in TNBC. By integrating this gene signature with HTS^2^ technology, we identified ponatinib as an effective agent that blocks MDSC infiltration into the cancer microenvironment, thus inhibiting the MDSC-mediated immunosuppression. These results strongly suggested that the gene signature-based high-throughput drug screening strategy is reliable and could be applied to identify potential drugs for other diseases.

Our findings clearly demonstrate that ponatinib reverses the immunosuppressive microenvironment by reducing MDSC infiltration. A previous study had shown that ponatinib modulates the immunosuppressive microenvironment in breast cancer by inhibiting hypoxia-inducible factor α (HIFα)-mediated PD-L1 expression in cancer cells [82]. However, our study uncovered a distinct mechanism in which ponatinib reshapes the immunosuppressive microenvironment by directly binding to and inhibiting p38 kinase activity in cancer cells, suppressing the STAT1–CXCL1/2 axis, and preventing MDSC infiltration. These findings highlight ponatinib as a potential MDSC-targeting compound for modulating the immunosuppressive microenvironment in solid cancers and lay the groundwork for the development of additional MDSC-targeting therapies.

This newly identified mechanism of action sheds light on the potential therapeutic effects of ponatinib in solid tumors. Although ponatinib is currently approved by the FDA for the treatment of leukemia, its immunomodulatory function within the TME has remained largely unexplored. In our 4T1 TNBC model, ponatinib significantly enhanced tumor sensitivity to anti-PD-L1 therapy, promoting the conversion of immunologically “cold” tumors into “hot” tumors. Collectively, our findings propose a novel chemoimmunotherapeutic strategy, in which combining ponatinib with PD-1/PD-L1 ICIs may offer enhanced clinical benefit in TNBC and potentially other cancers resistant to immunotherapy.

We provided compelling evidence that the inhibition of p38α, not SRC or ABL, suppresses the expression of *CXCL1* and *CXCL2* in TNBC, consequently hindering the recruitment of MDSCs. These findings indicate that inhibition of p38α by ponatinib not only attenuates the immunosuppressive functions of MDSCs but also impedes their migration, ultimately leading to potent antitumor effects. In addition to targeting p38α, we suggest that STAT1 might also represent another promising target for combinatory immunotherapy, warranting further investigation.

Our study emphasizes the therapeutic potential of ponatinib in remodeling the immunosuppressive TME by enhancing antitumor immunity through modulation of the p38α–STAT1–CXCL1/2 signaling axis and disrupting the function of immunosuppressive myeloid cells. These findings illustrate the multifaceted impact of ponatinib on both tumor and immune cell populations within the TME. Gaining a deeper understanding of these signaling dynamics is essential for designing innovative treatment strategies that exploit the role of immunosuppressive cell infiltration to improve immunotherapy outcomes. In our combination therapy experiments, ponatinib was administered for 2 weeks, while anti-PD-L1 treatment was given 4 times. This regimen led to a marked delay in tumor progression. However, further studies are needed to evaluate the long-term therapeutic efficacy of ponatinib and assess the potential emergence of drug resistance in breast cancer models.

In addition, prior research has demonstrated that silencing *CXCL1* and *CXCL2* significantly decreases the infiltration of CD11b^+^Gr1^+^ myeloid cells in lung metastases of breast cancer xenograft models [83]. These findings suggests that elevated expression of CXCL1 and CXCL2 is a key contributor to the metastatic progression of breast cancer. Our prior research also demonstrated that ponatinib inhibits breast cancer lung metastasis in mice through modulation of c-Jun [51]. Given our current findings that ponatinib suppresses 4T1 tumor growth by inhibiting the chemokines CXCL1 and CXCL2, we speculate that targeting the p38α–STAT1–CXCL1/2 axis is another way to limit disseminated tumor burden by ponatinib. Moreover, considering the important role of the CXCL1–CXCR2 axis in the pathogenesis of lung cancer [84], pancreatic ductal adenocarcinoma [57], and colorectal cancer [85], we hypothesize that the efficacy of ponatinib may extend beyond TNBC to other solid tumors.

The expression of chemokine varies across studies, reflecting the complexity of their regulation. Differences in tumor models [86], host immune status [87], tumor stage [88], and cytokine signaling [e.g., interferon-γ (IFN-γ)] [89] can all influence chemokine dynamics. Additionally, oncogenic drivers [90], epigenetic modifications [41], and immune pressure further shape expression patterns. Given their diverse roles in immune cell recruitment and activation [90,91], chemokines may exhibit divergent behaviors under different biological contexts. These factors help explain the inconsistent findings across studies and highlight the need for integrated interpretation.

Here, our integrated evidence highlights *CXCL1* and *CXCL2*, as well as tumor *MAPK14* and *STAT1* as promising clinical biomarkers. Transcriptomic analysis of 73,326 tumor samples revealed that elevated expression of these genes correlates with poor prognosis, supporting their role as prognostic markers. Their inclusion in compound-screening signatures further suggests utility as pharmacodynamic biomarkers for monitoring target engagement in clinical trials. Critically, validating the link between reduced *CXCL1*/*CXCL2* levels and intratumoral MDSC depletion would confirm a mechanistic basis for drug efficacy. This association also indicates that patients with high baseline *CXCL1*/*CXCL2* expression may be more likely to benefit from ponatinib.

In summary, our study leveraged gene signature-based drug screening to identify ponatinib as a potent modulator of the TME. We demonstrated that ponatinib suppresses CXCL1 and CXCL2 expression via the p38–STAT1 signaling axis, thereby limiting the recruitment of MDSCs into the TME and enhancing the efficacy of immunotherapy in TNBC. These findings offer a promising strategy for reshaping the immunosuppressive TME and highlight ponatinib as a potential combination agent to convert “cold” tumors into “hot” tumors, paving the way for improved immunotherapeutic responses.

## Materials and Methods

### Study design

We implemented our proprietary HTS^2^ approach [50,51] to screen against a compound library comprising 8,199 compounds, including FDA-approved therapeutics, epigenetic modulators, and bioactive natural products. The primary aim was to discover agents capable of regulating the expression of *CXCL1* and *CXCL2*, chemokines critically involved in MDSC recruitment to the TME. Candidate compounds were evaluated using RT-qPCR and Western blotting to quantify transcript and protein levels, respectively. Both in vitro and in vivo experiments were conducted using breast cancer and colorectal cancer models. Murine models included BALB/c, BALB/c nude, NSG, and C57BL/6 mice to comprehensively assess the impact of candidate compounds on MDSC infiltration and their potential to enhance anti-PD-L1 efficacy, thereby controlling tumor growth.

### Single-cell sequencing data analysis

We analyzed 2 publicly available scRNA-seq datasets from breast cancer studies, which included unique molecular identifier (UMI) count matrices, gene expression profiles, and corresponding cell barcodes, obtained in accordance with the procedures outlined in the original publications [65,71]. Cells were filtered based on quality control metrics: Those expressing fewer than 200 genes or exhibiting over 30% mitochondrial RNA content were excluded from further analyses [92]. Downstream processing was carried out using the Seurat (version 3.0) pipeline [93], following established workflows that encompassed object initialization, cell clustering, dimensionality reduction, and differential gene expression analysis via the FindMarkers function. Cell annotations incorporated ICB response classification: patients with T cell clonotype expansion (responders, Es) and those without marked expansion (nonresponders, NEs), as well as breast cancer molecular subtype (TNBC, HER2^+^, and ER^+^), as defined in the original study [65].

### MDSC identification

MDSCs were identified through a supervised machine learning approach utilizing the scPred package [70]. A reference breast cancer scRNA-seq dataset with well-defined MDSC populations [71] was used to train the classifier via the trainModel function. Model performance and cell type prediction confidence within the reference dataset were assessed using the get_probabilities function. Following model training, the classifier was applied to a separate breast cancer dataset containing ICB treatment data [65]. Cell type predictions, including MDSCs, were made using the scPredict function within the scPred framework [70].

### Gene signature collection and gene set enrichment

To facilitate visualization and comparative analysis of multi-gene signatures, we curated and analyzed 4 distinct gene sets. These included (a) MDSC-attracting chemokines: *CXCL1* and *CXCL2* [56,57]; (b) effector T cell markers: *GZMB*, *GZMK*, *PRF1*, and *IFNG* [94]; (c) immunosuppressive mediators of MDSCs: *IL1B*, *IL6*, *IL10*, *TGFB*, *VEGFA*, *CD274*, *ROS1*, *NOS2*, *IDO2*, *IDO1*, *ARG1*, *ARG2*, *ADAM17*, and *PTGES2* [74,75]; (d) a comprehensive panel of MDSC-associated genes derived from prior studies [71,79,80]: *S100A8*, *S100A9*, *IFR1*, *CD33*, *CD34*, *CD38*, *HLA-DR*, *CD11B*, *CD15*, *CD66B*, *CD14*, *CSF-1R*, *STAT1*, *STAT3*, *STAT6*, *NF-KB*, *C-EBP-B*, *c-Rel*, *NP63*, *IFR8*, *ANXA1*, *LYZ2*, *CD244*, *CD36*, *CD84*, *DR5*, *CXCR1*, *FATP2*, *LOX1*, *PDL1*, *WFDC17*, *IL4R*, *OLR1*, *IFITM1*, *IL1B*, *PROK2*, *IFITM2*, *JUNB*, *DUSP1*, *SOCS3*, *BTG1*, *SELPLG*, *ASPRV1*, *IGFBP6*, *PLA2G7*, *CSF3R*, *CXCR2*, *TPD52*, *TSPO*, *GRINA*, *FABP5*, *CLEC4D*, *STEAP4*, *MAP1LC3B*, *CCR1*, *LRG1*, *CLEC4E*, *CTSD*, *GCNT2*, *ARG2*, *NPL*, *FGL2*, *RNF149*, *SEPHS2*, *S100A6*, *LMNB1*, *EIF4EBP1*, *MSRB1*, *UBB*, *C5AR1*, *YPEL3*, *GSR*, *TALDO1*, *ATP6V1G1*, *S100A11*, *HP*, *ALOX5AP*, *LITAF*, *UPP1*, *GPCPD1*, *SNAP23*, *HIST1H2BC*, *MYD88*, *ADIPOR1*, *STK17B*, *ZYX*, *RGS3*, *HDC*, *ATG3*, *GDA*, *SLC40A1*, *CDK2AP2*, *GLIPR2*, *TACSTD2*, *PICALM*, *MTUS1*, *FBXL5*, *RND1*, *IER2*, *MXD1*, *CDKN2D*, *SELL*, *PPT1*, *SKAP2*, *SFXN5*, *ATP11B*, *CXCL2*, *OSM*, and *IER3.* For each cell, enrichment scores corresponding to these gene modules were computed using the AddModuleScore function from the Seurat package [93]. Feature plots were generated using Seurat (version 3.0) [93], while box plots were created with GraphPad Prism (version 9.0) to visualize the distribution and comparative expression patterns of gene signatures across cell populations.

### Comparative analysis of *CXCL1*/*CXCL2* high and low expression groups

*CXCL1* and *CXCL2* signature scores, along with MDSC scores, were computed using the AddModuleScore function in Seurat [93], based on the curated gene sets described previously. Cell type annotations were generated using the scPred [70] package as detailed above. To stratify the dataset, individual cells and patients were categorized into *CXCL1/CXCL2* high or low groups, based on the average combined expression scores of *CXCL1* and *CXCL2*. MDSC score comparisons were then performed across these groups at 3 levels: (a) single cell level—comparing the distribution of MDSC scores across all individual cells; (b) cell type level—averaging MDSC scores within each annotated cell type; (c) patient level—calculating the mean MDSC score for each patient, either across all cells or within a specific immune cell subset.

Additionally, the relative abundance of each cell type was quantified as the proportion of total cells assigned to that type. These proportions were then compared between *CXCL1/CXCL2* high and low groups to assess differences in immune landscape composition, with a particular focus on MDSC infiltration and shifts in immune cell distribution associated with *CXCL1*/*CXCL2* expression profiles.

### Correlation analyses

Correlation analyses were conducted at both the tumor cell and patient levels using scRNA-seq data from a breast cancer cohort and bulk RNA-seq data from the TCGA BRCA dataset. At the single-cell level, *CXCL1* and *CXCL2* gene signature scores were computed for individual tumor cells as previously described. For each patient, the mean expression levels of *CXCL1* and *CXCL2* within tumor cells were correlated with the expression levels of key MDSC-associated immunosuppressive genes [74,75]. These correlations were computed using the cor function in R. Scatterplots were generated to visualize these correlation results and the expression levels of *CXCL1* and *CXCL2*, as well as each immunosuppressive molecule [74,75], using GraphPad Prism version 9.0. At the patient level, we correlated the average expression of *CXCL1* and *CXCL2* (aggregated across all tumor cells per patient) with the average expression of T cell activation markers (e.g., GZMB) computed across all T cells within the same patient. This analysis included data from 29 individual breast cancer patients. Correlation coefficients were again calculated using R, and corresponding scatterplots were produced in GraphPad Prism to illustrate interpatient relationships between tumor-derived chemokines and T cell effector function. In addition, pan-cancer correlation analysis was performed using data from 34 tumor types in the TCGA cohort. Specifically, expression levels of CXCL1 and CXCL2 were correlated with MDSC-associated immunosuppressive markers [74,75] using the Tumor Immune Estimation Resource (TIMER 2.0) platform [72], enabling cross-cancer evaluation of chemokine–immunosuppressive gene associations.

### IHC of TNBC tissue microarray

A human TNBC tissue microarray (catalog no. HBreD129Bc02-1, Shanghai Outdo Biotech, China), comprising 129 tumor samples from 129 patients, was used to assess CXCL2 and CD33 expression on 2 consecutive sections (Table S3). IHC was performed by Outdo Biotech following standard protocols [95]. Hematoxylin and eosin (H&E) staining confirmed pathological classification. Primary antibodies against CXCL2 (1:500, catalog no. 16325-1-AP, Proteintech) and CD33 (1:500, catalog no. 84341-3-RR, Proteintech) were applied. Protein expression was quantified using the H-score method [95], and statistical comparisons were performed using the chi-square test. Representative IHC images from 4 TNBC cases were visualized with Aperio ImageScope (v12.4.6), and quantitative analysis was conducted using HALO (Indica Labs, USA).

### ST data analysis

Publicly available Visium ST data from one breast cancer tissue section were obtained from the Gene Expression Omnibus (GEO) database (accession code: GSE243280). Raw spatial gene expression matrices were processed and visualized using Seurat (v5). Data were normalized and log-transformed prior to downstream analyses. The SpatialFeaturePlot function was used to visualize the spatial expression patterns of key genes, including *CXCL1*, *CXCL2*, *STAT1*, and *MAPK14*. Tumor cell-enriched regions were identified based on histological annotations and gene expression profiles from the original study, and manually highlighted on the tissue slide images. For colocalization analysis, expression values of *STAT1* and *MAPK14* were extracted per spatial spot. Coexpression was defined as the overlapping presence of both transcripts in the same spatial location. Composite spatial plots were generated to display *MAPK14* (red), *STAT1* (blue), and their coexpression (fuchsia) within the tissue section.

### Performance of *CXCL1* and *CXCL2* in predicting ICB response

To assess the predictive potential of *CXCL1* and *CXCL2* gene signatures for ICB response, we analyzed 1 breast cancer scRNA-seq dataset [65] and 9 publicly available bulk RNA-seq datasets comprising a total of 300 patients across multiple cancer cancer types, including melanoma [96–102], glioblastoma [103], NSCLC [104–106], and head and neck squamous carcinoma (HNSC) [107] (Table S1). Four of these bulk datasets were acquired from the TIDE website [96–98,108].

For each dataset, raw RNA-seq counts were normalized and subjected to *z* score transformation by subtracting the mean expression across all samples. To evaluate differences between responders (R) and nonresponders (NR) to ICB treatment, we conducted unpaired, nonparametric *t* tests. Box plots were generated using GraphPad Prism (v9.0) to illustrate the *z* score distributions for the *CXCL1* and *CXCL2* signatures across different cohorts and cancer types. We further benchmarked the predictive accuracy of the *CXCL1*/*CXCL2* signature against established ICB response biomarkers using tools provided by the TIDE online platform [109], applying this comparative analysis across the 9 bulk RNA-seq datasets (Table S1).

Furthermore, to assess the relationship between the average expression level of *CXCL1*-*CXCL2* and MDSC-related genes with ICB response at the single-cell level, we utilized the scRNA-seq matrix of breast cancer (obtained from the original study [65]). At the single-cell level, the average expression of *CXCL1* and *CXCL2* in tumor cells from R and NR of breast cancer patients was calculated. Like the bulk RNA-seq analysis, an unpaired, nonparametric *t* test was used to compare the groups of R and NR. The results were visualized using box plots to display the average expression levels of *CXCL1* and *CXCL2*, as well as MDSC-related genes in breast cancer, using GraphPad Prism version 9.0.

### Clinical outcome analyses

To investigate the relationship between the average expression of *CXCL1* and *CXCL2* and OS in patients, we analyzed multiple datasets containing ICB therapy information. These datasets were accessed from the TIDE website [109] and GEO [110–112] across the 9 datasets (Table S1). Kaplan–Meier (KM) plots for *CXCL1* or *CXCL2* were also generated on other platforms, including cBioPortal [109]. The model provided the Rho values, which represent the prognosis of patients based on the expression levels of *CXCL1* and *CXCL2*. Patients were stratified into high or low expression subgroups for *CXCL1* and *CXCL2*, and the KM survival plots were generated. The statistical significance of survival differences was determined using a log-rank test on the TIDE website [109]. Additionally, we compared the clinical outcome prediction performance of the *CXCL1* and *CXCL2* signatures with other established biomarkers using the TIDE online tool [109] across the 9 datasets (Table S1). KM plots for *CXCL1* or *CXCL2* were also generated on other platforms, including cBioPortal [110], TIMER [72], and a web-based KM plotter [113], using datasets from breast cancer patients.

We analyzed the genomic alterations of *CXCL1* and *CXCL2* using data from the cBioPortal [110] website. The analysis included gene alteration information for all patients across breast cancer datasets (pan-BRCA) as well as across all cancer types (pan-cancer).

### Publicly available datasets

In this study, we performed analyses using datasets obtained from the following sources: (a) 2 scRNA-seq datasets (1: *n* = 29, 1 cohort, 175,942 cells, 2: PyMT mice, 14,646 cells), (b) 1 Visium ST data from breast cancer tissue sections [68] from the GEO (*n* = 1, 1 cohort), (c) bulk RNA-seq dataset [67] from CPTAC (*n* = 122, 1 cohort), (d) 17 bulk RNA-seq datasets from TIDE (*n* = 1,180, 17 cohorts), and (e) 40 bulk RNA-seq datasets from cBioPortal (TCGA, *n* = 70,655, 217 cohorts) (Table S1). In total, the analyses incorporated data from 190,588 single cells, 238 cohorts, and 73,326 cancer patients across 34 cancer types (Table S1).

### Chemicals

The drug screening library contained 8,199 compounds, systematically organized into 3 distinct categories: FDA-approved therapeutic compounds, epigenetic modulators, and bioactive natural products. Within this repository, 1,154 clinically approved drugs were procured from Selleck Chemicals. The remaining chemicals were sourced from the Shanghai Institute of Materia Medica. Ponatinib was purchased from TargetMol. SB 203580 and SB 239063 were purchased from MedChemExpress. All chemicals were dissolved in dimethyl sulfoxide (DMSO) for in vitro studies.

### Cell culture

The MDA-MB-231, 4T1, LM2, MCF7, SW480, SW620, HCT116, HeLa, and A549 cell lines were obtained from the Institute Cancer Cell Line Encyclopedia of China. The MC38 cell line was kindly provided by D. Pan from Tsinghua University. Cells were cultured in Dulbecco’s modified Eagle’s medium (DMEM) (Gibco) supplemented with 10% fetal bovine serum (FBS) (Gemini) and 1% penicillin/streptomycin (Gibco). Cells were cultured at 37 °C in a 5% CO_2_ atmosphere.

### Plasmids and antibodies

Lentiviral shRNAs were provided by the Vector Core at Tsinghua University. The following antibodies from Cell Signaling Technology were used in this study at a dilution of 1:1,000: anti-p38 (#9212S), anti-p-p38-Thr^180^/Tyr^182^ (#4511S), anti-STAT1 (#14994S), anti-p-STAT1-Tyr^701^ (#7649T), anti-p-STAT1-Ser^727^ (#9177S), anti-p-ERK1/2 (#4370T), anti-ERK1/2 (#4695T), anti-p-JNK (#4668S), anti-JNK (#9252S), anti-GAPDH (glyceraldehyde-3-phosphate dehydrogenase) (#2118S), and anti-tubulin (#2128S).

### Drug screening and enrichment score calculation

The pharmacological screening dataset employed in this study was generated by Shao et al. [51]. Our computational pipeline for quantifying compound efficacy in down-regulating *CXCL1* and *CXCL2* expression was adapted from the Connectivity Map framework [49] and our previous work [43,51,58].

Approximately 3,000 MDA-MB-231 cells were seeded per well in 384-well plates and, after 24 h, treated with small molecules from the screening library at a concentration of 1 μM for 24 h. Library preparation and sequencing followed the protocol described by Li et al. [114] . mRNA levels were quantified by high-throughput sequencing on the HiSeq 2000 platform. The gene signature was derived from a curated collection of chemokine-related genes associated with cold tumors [14,15].

For data processing, sequencing reads were mapped to probe sequences (allowing up to 3 mismatches) and normalized to the expression of 33 stable breast cancer genes [115]. Batch effects were assessed by principal components analysis (PCA) using the FactoMineR [116] and factoextra (https://github.com/kassambara/factoextra) tools and corrected using the ComBat [117] algorithm. For each compound, fold-change values were calculated relative to the averaged DMSO controls on the same plate. Compounds were ranked by their ability to down-regulate *CXCL1* and *CXCL2*, and significance was assessed by Student’s *t* test, with *P* values and fold changes used for prioritization.

Randomization was applied during library preparation and sequencing, and investigators remained blinded to clinical outcomes throughout computational analyses. Primary and secondary endpoints were predefined to ensure transparency and reproducibility. Sample size was determined by statistical power calculations using the R package pwr (http://cran.r-project.org/web/packages/pwr/), designed to detect clinically meaningful differences between groups.

### Mice and reagents

All animal protocols (#19-WD1 and #19-WD1-1) used in this study were approved by the Institutional Animal Care and Use Committee (IACUC) of Tsinghua University and were conducted in accordance with guidelines established by the Association for Assessment and Accreditation of Laboratory Animal Care International (AAALAC).

Five- to 6-week-old female BALB/c WT mice, BALB/c nude mice, and C57BL/6 WT mice were commercially procured from Vital River Laboratory Animal Technology. NSG mice were obtained from The Jackson Laboratory. Mice were housed in the animal facility of the Laboratory Animal Research Center of Tsinghua University under specific pathogen-free (SPF) conditions. Briefly, subconfluent 4T1 cells (5 × 10^4^) were suspended in 0.04 ml of DMEM medium and injected into the mammary fat pads of BALB/c WT mice, BALB/c nude mice, and NSG mice under anesthesia using avertin. For the MC38 tumor model, C57BL/6 female mice were subcutaneously injected in the right flank with 1 × 10^6^ MC38 tumor cells. When tumors reached a volume of approximately 100 mm^3^, mice were randomized based on tumor volume into various treatment groups. The dosing regimen was adopted from previously published studies in which ponatinib (30 mg/kg) or vehicle was administered orally for 3 weeks, showing pronounced antitumor efficacy with tolerable toxicity profiles [51,118].

For combination therapy, ponatinib treatment was administered for 2 weeks, in combination with an intraperitoneal injection of either an anti-PD-L1 antibody (10 mg/kg twice weekly for 2 weeks, a total of 4 injections; clone 10F.9G2, BioXcell) [119–121] or an isotype control antibody (10 mg/kg twice weekly for 2 weeks, total of 4 injections; clone LTF2, BioXcell).

Tumor volumes were measured using digital calipers and calculated with the following formula: volume (mm^3^) = [width^2^ (mm^2^) × length (mm)]/2. After 22 days of treatment, mice were sacrificed and tumors were isolated for further analysis by fluorescence-activated cell sorting (FACS).

### MDSCs mixed with 4T1 cells for transplantation

4T1 cells (5 × 10^4^) were mixed with sorted MDSCs (1 × 10^5^) isolated from 4T1 tumor-bearing mice, and the mixture was injected into the mammary gland of recipient mice. Tumor growth curves and tumor weight were recorded throughout the experiment to assess the effects of MDSC cotransplantation on tumor progression.

### MDSC isolation and in vitro migration assay

MDSCs were isolated from the spleens of 4T1 tumor-bearing mice using the Mouse MDSC Isolation Kit (Miltenyi Biotec, catalog no. 130-094-538). The isolated cells were plated in RPMI 1640 medium supplemented with 10% FBS and antibiotics for subsequent assays. MDSCs (1 × 10^5^ cells per well) were seeded into the top chamber of a transwell (Corning). CM from cultured 4T1 or MC38 cells, treated with 1 μM ponatinib or DMSO for 48 h, were collected and supplemented with either vehicle or recombinant mouse CXCL1 (100 ng/ml) and CXCL2 (10 ng/ml). The CM was added to the bottom chamber of the transwell. After 6 h of incubation, the cells that had migrated to the bottom chamber were counted. These experiments were performed in triplicate, and statistical significance was assessed using Student’s *t* test.

### Flow cytometry analysis

Splenocytes and tumor-infiltrating lymphocytes (TILs) were isolated, and red blood cells were lysed. The single-cell suspensions were incubated with anti-mouse CD16/32 (BioLegend) for 10 min at 4°C to block nonspecific binding. After blocking, the cells were stained with the following antibodies for 40 min on ice: anti-mouse CD4 Super Bright 600 (eBioscience, #63-0042-80), anti-mouse CD8a phycoerythrin (PE)-eFluor 610 (eBioscience, #61-0081-80), anti-mouse CD3e peridin chlorophyll protein (PerCP)-Cyanine 5.5 (eBioscience, #45-0031-80), anti-mouse CD3e PE-Cyanine 5 (eBioscience, #15-0031-82), anti-mouse CD45 eFluor 450 (eBioscience,#48-0451-80), anti-mouse CD45 Super Bright 780 (eBioscience, #78-0451-82), anti-mouse Ly-6G/Ly-6C PE-Cyanine7 (eBioscience, #25-5931-82), anti-mouse Ly-6G-PE (eBioscience, #12-9668-80), anti-mouse Ly-6C eFluor 450 (eBioscience, #48-5932-80), anti-mouse F4/80 PE-Cyanine 7 (eBioscience, #25-4801-82), anti-mouse F4/80 fluorescein isothiocyanate (FITC) (eBioscience, #11-4801-81), anti-mouse CD11b FITC (eBioscience, #11-0112-41), and anti-mouse CD11b eFluor 506 (eBioscience, #69-0112-80). Flow cytometry was performed using the FACSAria III flow cytometer (BD Biosciences), and the data were analyzed using FlowJo software.

### RT-qPCR

Total RNA was extracted using TRIzol (Invitrogen) and used for cDNA synthesis with the Thermo Fisher Scientific RevertAid First Strand cDNA Synthesis Kit (Thermo Fisher Scientific). RT-qPCR was performed using a StepOne Plus real-time PCR system (KAPA) to quantify gene expression levels.

### ELISA

Cells (2 × 10^6^/100-mm dish) were cultured for 24 h. The media were then replaced with 10 ml of fresh DMEM containing different concentrations of ponatinib or DMSO. Supernatants were collected at 24 or 48 h, with floating cells removed by 0.45-μm filtration. The amount of CXCL1 or CXCL2 protein in the supernatant was measured using a mouse CXCL1- or CXCL2-specific ELISA kit (R&D Systems), according to the manufacturer’s instructions.

### Immunoblotting

Cells were lysed with radioimmunoprecipitation assay (RIPA) lysis buffer containing 1 mM phenylmethylsulfonyl fluoride (PMSF) and a 1× protease inhibitor cocktail (MedChemExpress). Protein concentration was quantified using the bicinchoninic acid (BCA) assay (Thermo Fisher Scientific). Protein samples were separated by sodium dodecyl sulfate–polyacrylamide gel electrophoresis (SDS-PAGE) and transferred to polyvinylidene difluoride (PVDF) membranes (Bio-Rad). Membranes were probed with antibodies, as indicated in the figure legends. Protein bands were visualized using SuperSignal West Pico reagent (Thermo Fisher Scientific). GAPDH or tubulin was used as an internal control.

### RNA-seq library construction

MDA-MB-231 cells (1 × 10^6^) were plated in a 10-cm plate for 24 h and then treated with ponatinib at 1 μM for 24 h. Similarly, 2 × 10^6^ 4T1 cells were plated in a 10-cm plate for 24 h and treated with ponatinib at 1 μM for 24 h. Cells were harvested, and total RNA was isolated using TRIzol (Invitrogen). RNA libraries were constructed using the NEBNext Ultra RNA Library Prep Kit for Illumina (New England Biolabs) and sequenced on the NovaSeq6000 sequencing system (Illumina).

### RNA-seq data analysis

Clean reads were obtained after removing adapter sequences and poly-N reads. These reads were aligned to the reference genome *Homo sapiens* (hg38) and *Mus musculus* (GRCm39) [122] using HISAT2 [123]. Gene read counts were obtained by assembling transcripts with HTSeq [124] for each sample. Differentially expressed genes (DEGs) were identified using DESeq2, with a significance threshold of a *P* value < 0.05 and |fold change| ≥ 1.5.

### GO analysis and GSEA

GO analysis was performed on genes showing decreased expression [log_2_(fold change) < −1 and *P* < 0.05] in ponatinib-treated cells using the clusterProfiler package [125]. GSEA was conducted on ponatinib-treated versus DMSO-treated MDA-MB-231 or 4T1 cells using the GSEA tool (version 4.3.2) in clusterProfiler [125], with the “PreRanked” mode. The normalized enrichment score (NES), nominal *P* value, and false discovery rate (FDR) were calculated with 1,000 permutations using the Signal2Noise metric.

### Molecular docking

The crystallographic structure of human p38α MAP kinase was retrieved from the Protein Data Bank (https://www.rcsb.org/). Structural preprocessing was performed using Schrödinger’s Protein Preparation Wizard (Schrödinger Release, 2023-1). Schrödinger’s Receptor Grid Generation module was utilized to construct a 3-dimensional grid system encompassing the native ligand, thereby delineating the ligand-binding pocket through spatial coordinate parameterization. Ligand–receptor docking simulations were conducted with Schrödinger’s Glide module, followed by molecular mechanics/generalized born surface area (MM/GBSA) binding free energy calculations. Ligand–receptor interactions were visualized using the Ligand Interaction Diagram module.

### p38α kinase assay

The p38α kinase assay was conducted following the manufacturer’s instructions. Reactions were prepared in a 25-μl mixture containing 1× Reaction Buffer A, 1% DMSO, 50 μM dithiothreitol (DTT), 10 μM adenosine triphosphate (ATP), 40 ng of p38α kinase, 1 μg of p38α peptide substrate, and serial dilution of ponatinib. For the control, the same titration was performed with all the reaction components except the enzyme. The reactions were incubated at room temperature for 60 min. After incubation, 25 μl of Kinase-Glo Reagent was added to each reaction, and the plate was incubated at room temperature for an additional 40 min. Kinase Detection Reagent was then added, and the plate was incubated for 30 min at room temperature. Luminescence was measured, and IC_50_ (median inhibitory concentration) values were determined.

### Statistical analysis

Statistical comparisons between 2 groups were performed using 2-way analysis of variance (ANOVA) and 2-tailed unpaired Student’s *t* tests. Log-rank tests were employed for KM survival analysis. Significance levels were defined as follows: *P < 0.05, **P < 0.01, ***P < 0.001, and ****P < 0.0001.

## Data Availability

Datasets used in this study are available from the publications referenced above and Table S1. All raw and processed RNA-seq data for ponatinib-treated cells have been deposited in the GEO database under the accession number GSE228550. The codes used in this study followed the manuals of each R package, all of which are fully open access. All software is either freely or commercially available. Further information and any requests for resources and reagents is available from the lead contact upon request.

## Supplementary Materials

Supplementary 1 Figs. S1 to S9 Tables S1 to S3 References [126–129]
