# Supplementary material for: Gene Signature-Based Drug Screening Reveals Ponatinib Enhances Immunotherapy Efficacy in Triple-Negative Breast Cancer by Reversing MDSC-Mediated Immunosuppressive Tumor Microenvironment
Source: Research (Wash D C). 2025 Oct 9;8:0915. doi: 10.34133/research.0915 (PMC12508528; doi:10.34133/research.0915)
Supplement: Supplementary 1 — Figs. S1 to S9 Tables S1 to S3 References [126–129] [file research.0915.f1.zip › Supplementary figure_20250907.pdf]

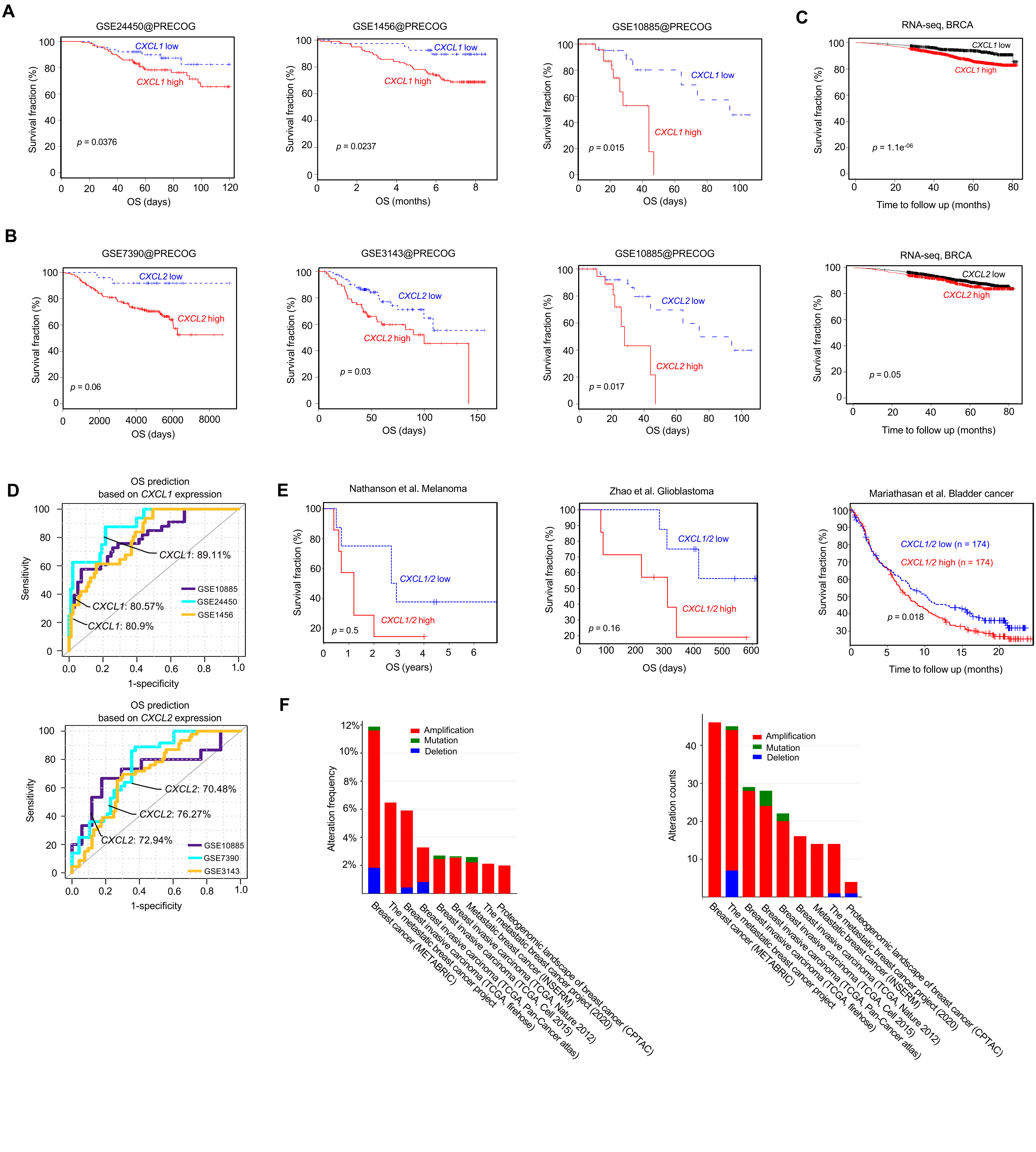

Figure S1. **The expression of *CXCL1* and *CXCL2* is associated with poor outcomes in pan-cancer**

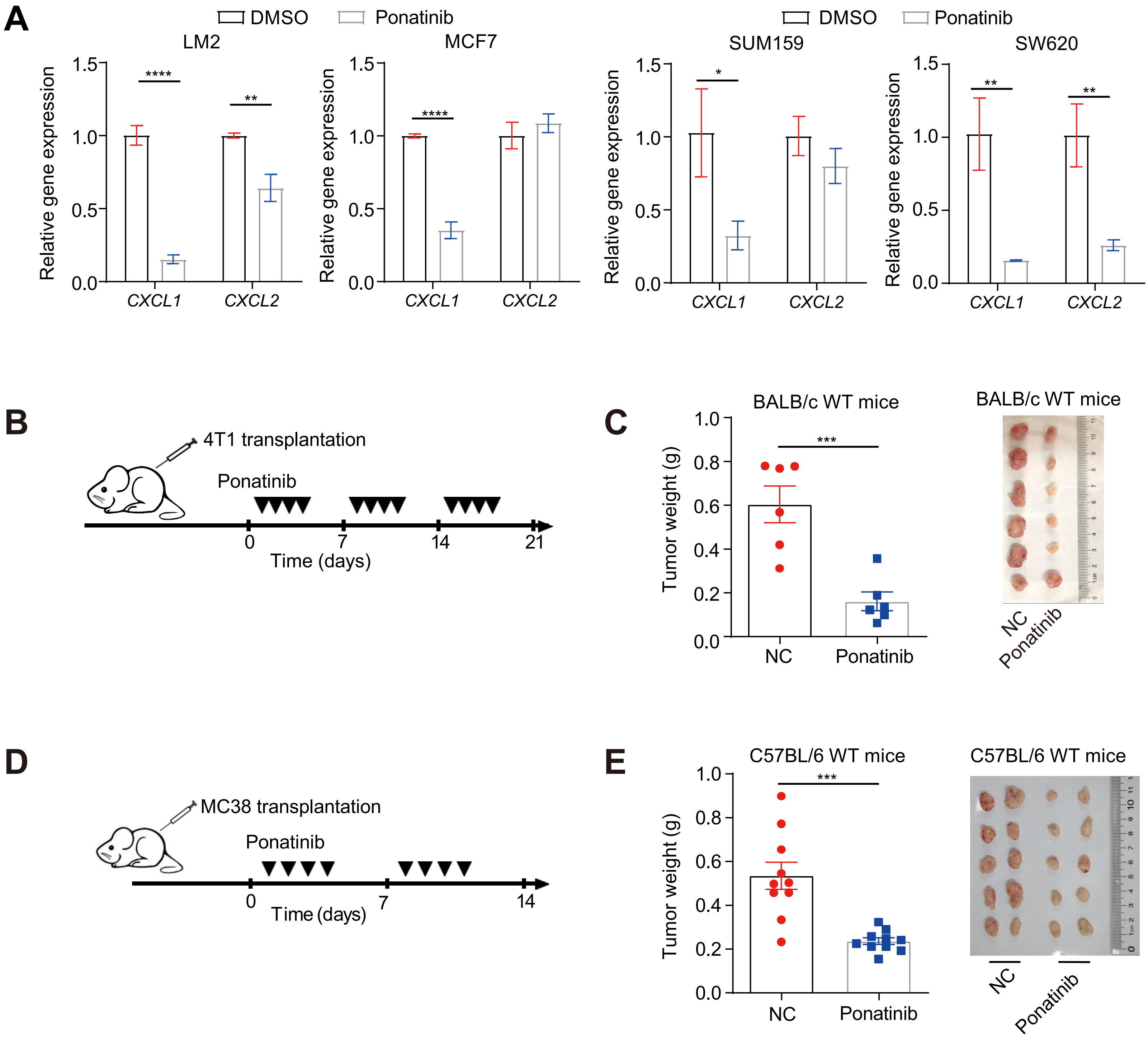

Figure S2. Ponatinib suppresses *CXCL1/2* expression in diverse cancer cells and inhibits 4T1 and MC38 tumor growth *in vivo*

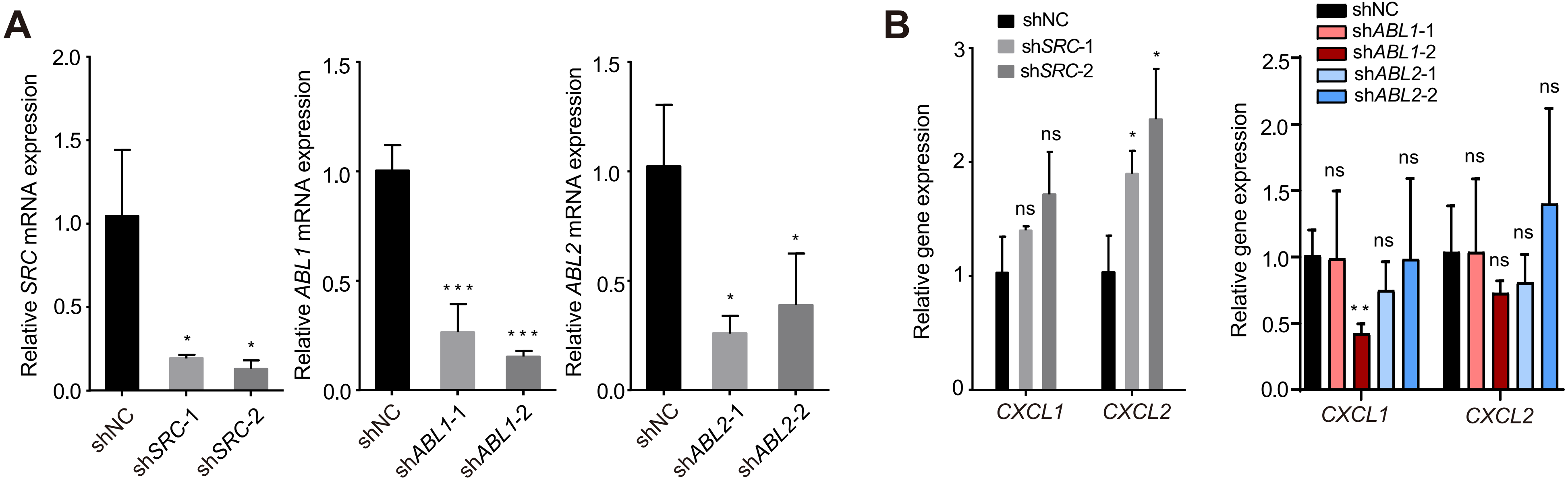

Figure S3. **SRC/ABL1/ABL2 are not the targets for the inhibition of *CXCL1* and *CXCL2* expression in TNBC cells by ponatinib**

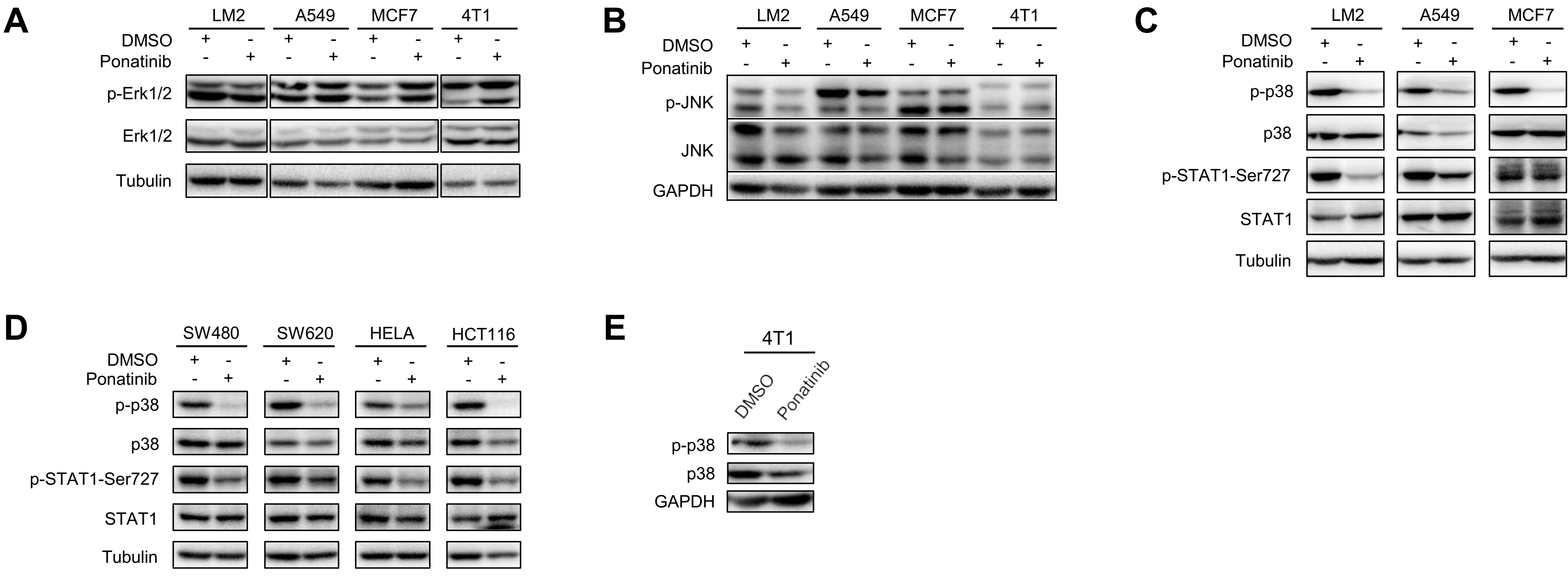

Figure S4. Ponatinib downregulates *CXCL1* and *CXCL2* transcription through p38 dephosphorylation

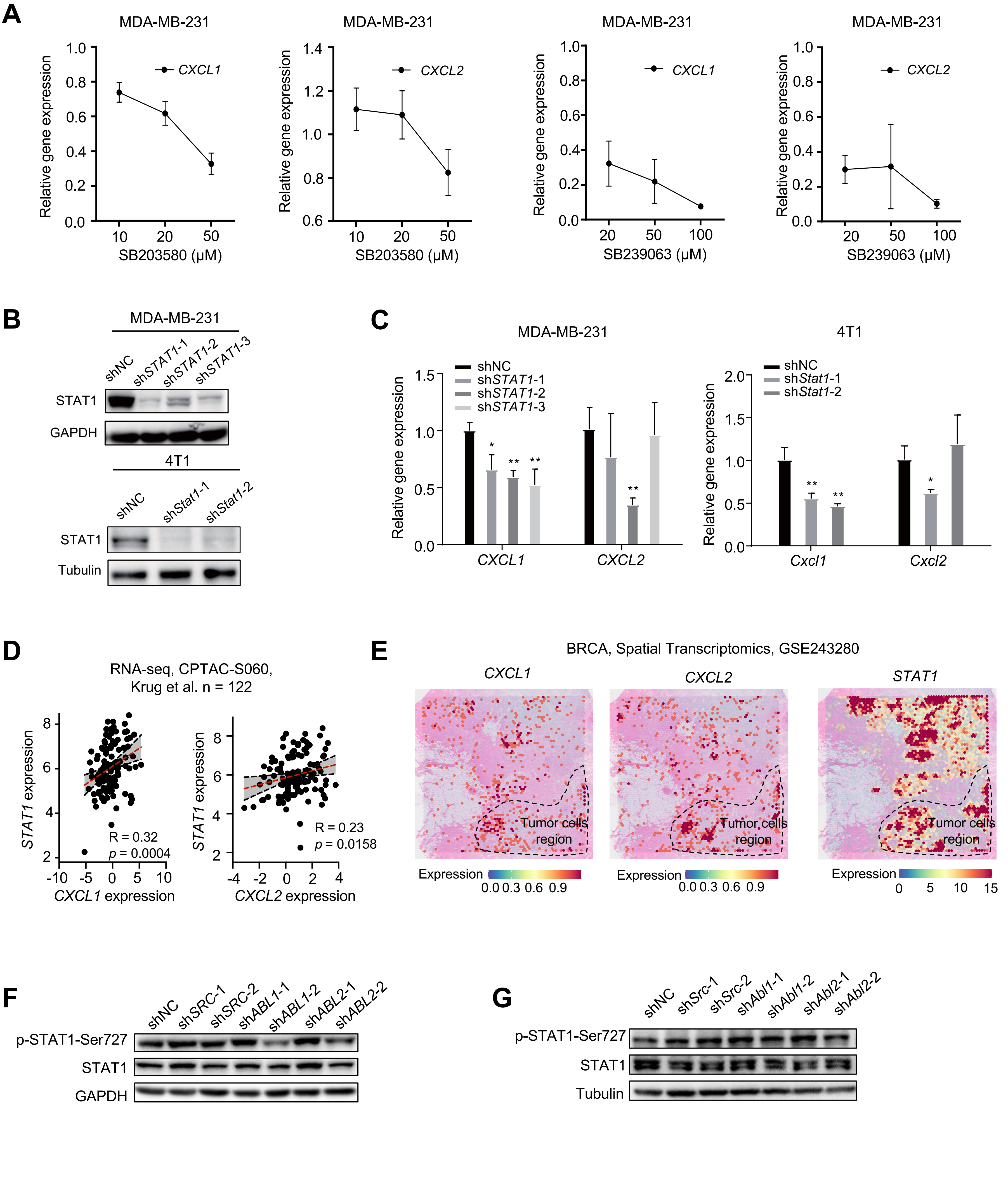

Figure S5. **STAT1 loss-of-function suppresses the expression of *CXCL1* and *CXCL2***

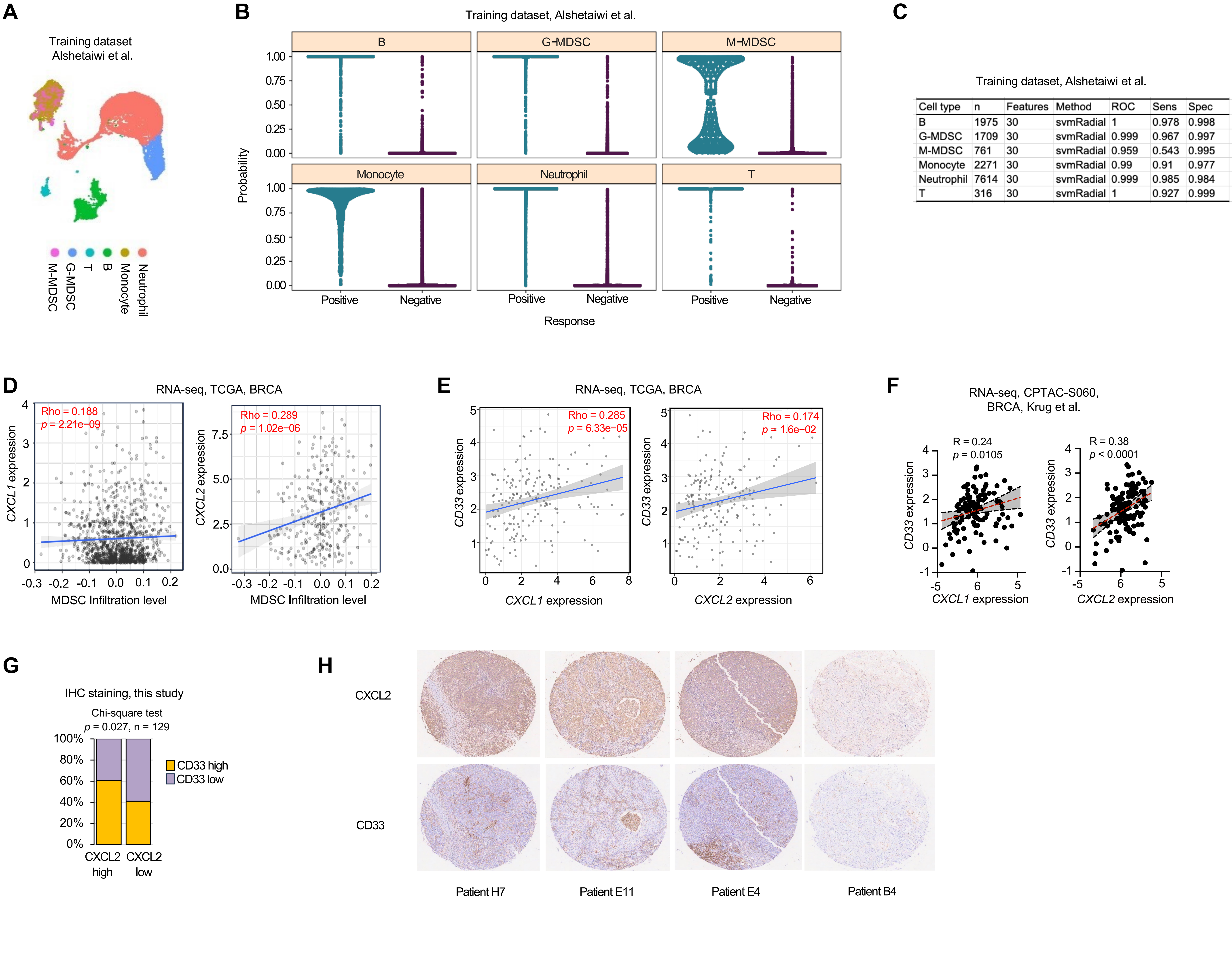

Cell type	n	Features	Method	ROC	Sens	Spec
B	1975	30	svmRadial	1	0.978	0.998
G-MDSC	1709	30	svmRadial	0.999	0.967	0.997
M-MDSC	761	30	svmRadial	0.959	0.543	0.995
Monocyte	2271	30	svmRadial	0.99	0.91	0.977
Neutrophil	7614	30	svmRadial	0.999	0.985	0.984
T	316	30	svmRadial	1	0.927	0.999

Figure S6. Higher MDSC frequencies are correlated to increased breast cancer progression

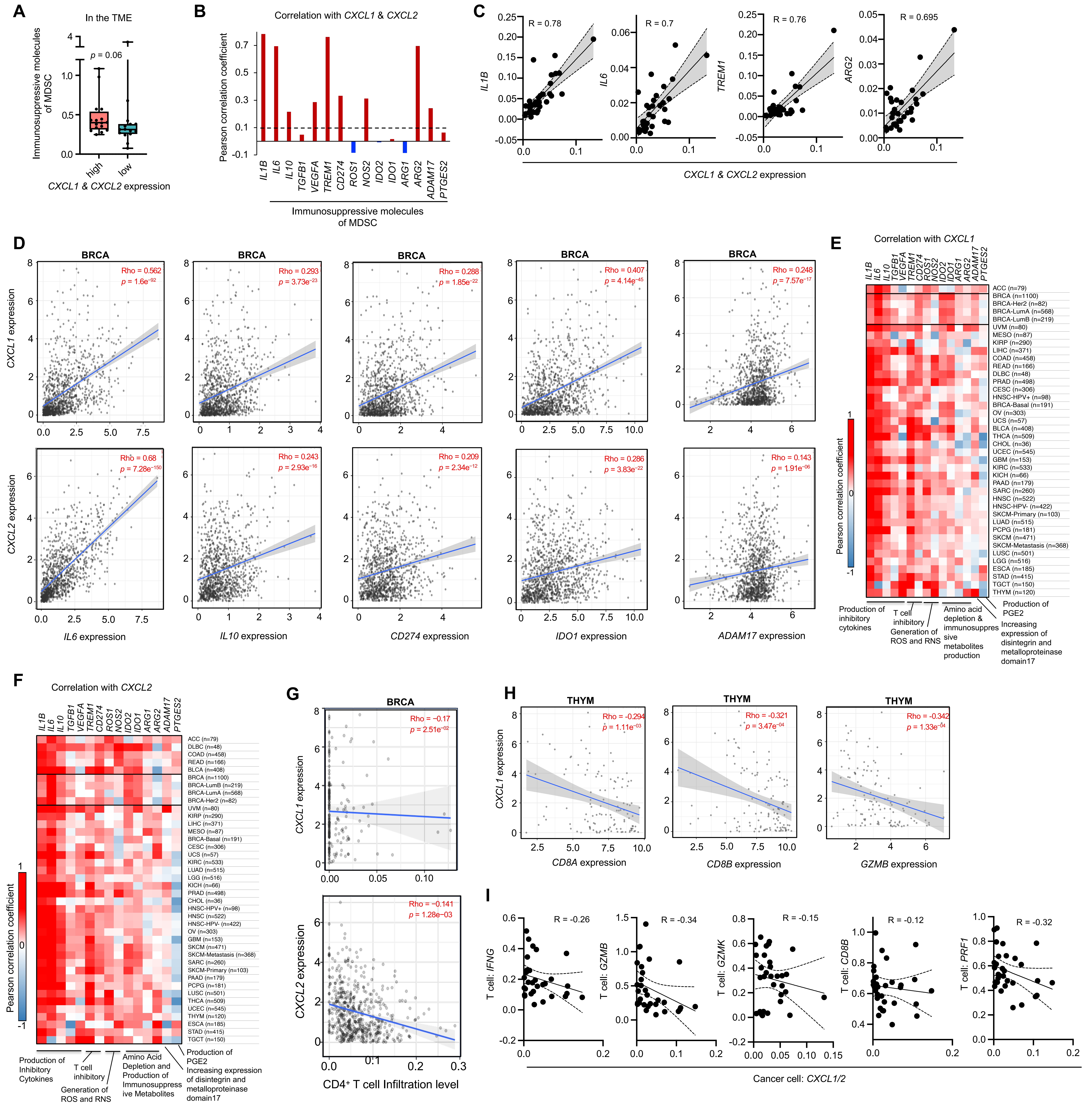

Figure S7. The expression of *CXCL1* and *CXCL2* in tumor cells is related to MDSC infiltration and an immunosuppressive TME

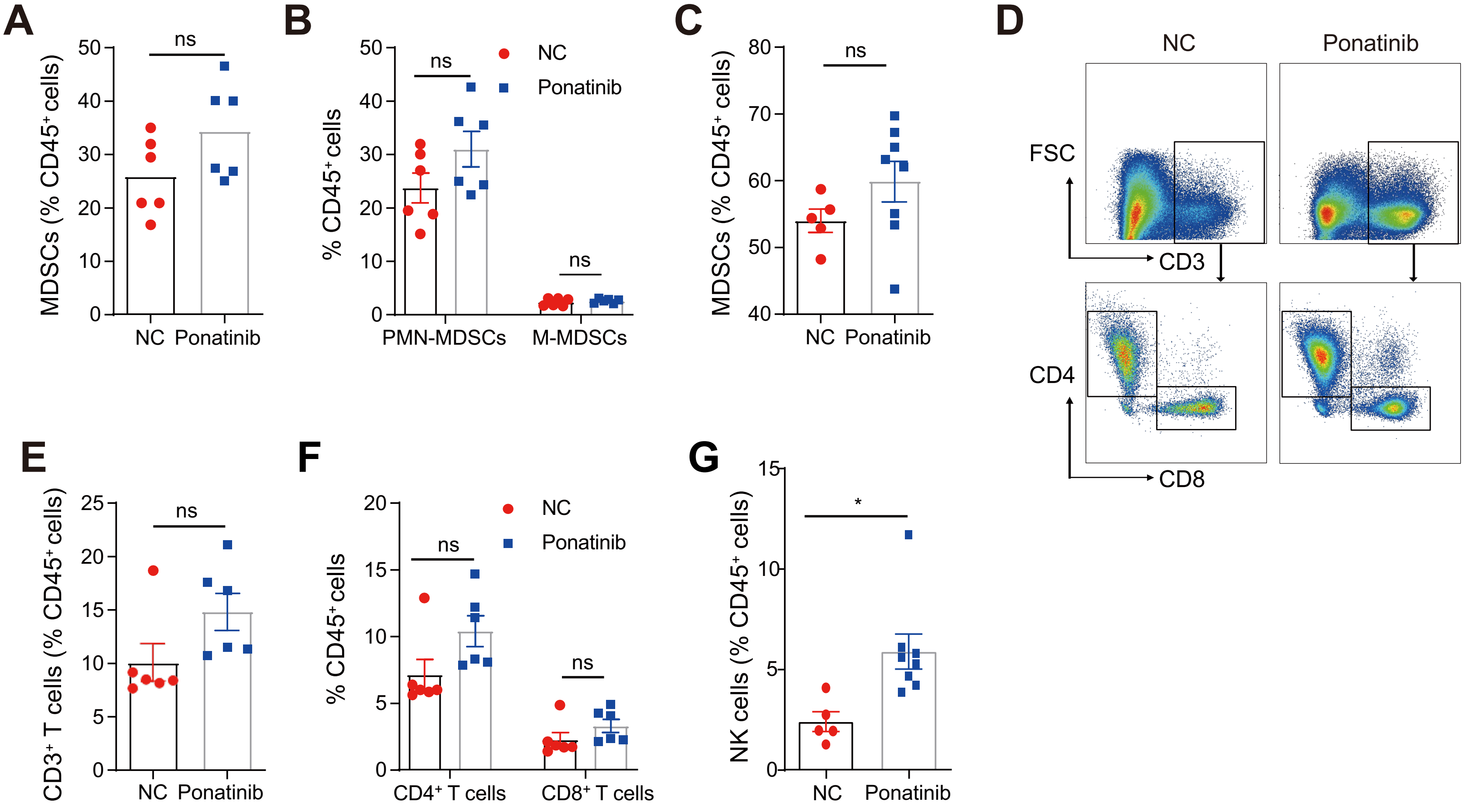

Figure S8. **Ponatinib slightly affects the distribution of immune cells in the spleen *in vivo***

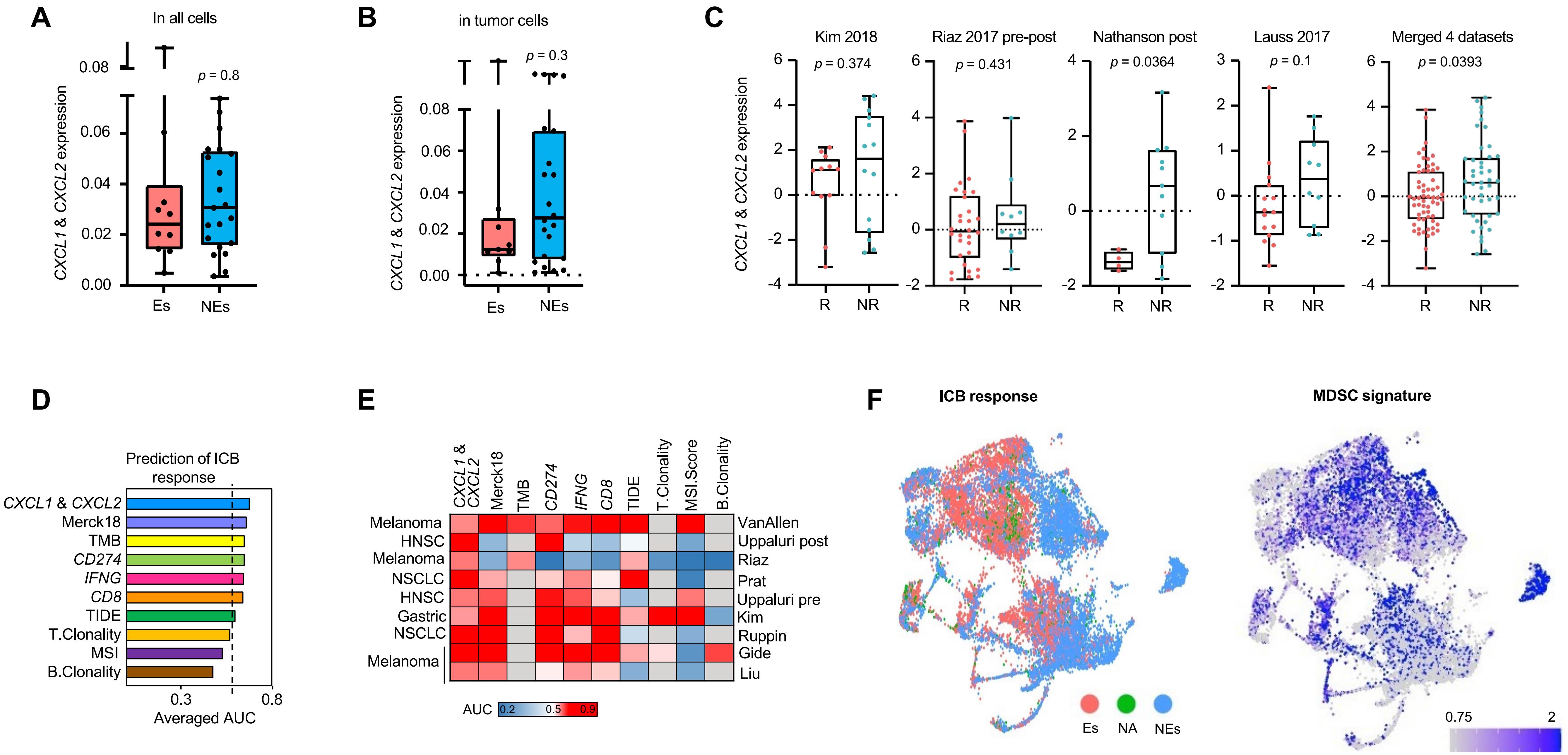

Figure S9. **The expression levels of *CXCL1* and *CXCL2* predict the response of immunotherapy**
