# Supplementary material for: Gene Signature-Based Drug Screening Reveals Ponatinib Enhances Immunotherapy Efficacy in Triple-Negative Breast Cancer by Reversing MDSC-Mediated Immunosuppressive Tumor Microenvironment
Source: Research (Wash D C). 2025 Oct 9;8:0915. doi: 10.34133/research.0915 (PMC12508528; doi:10.34133/research.0915)
Supplement: Supplementary 1 — Figs. S1 to S9 Tables S1 to S3 References [126–129] [file research.0915.f1.zip › supplementary_captions.docx]

**Supplemental information**

**Fig. S1. The expression of *CXCL1* and *CXCL2* is associated with poor outcomes in pan-cancer**

**(A-B)** Kaplan-Meier (KM) plots of OS for patients with breast cancer, grouped by high or low expression of *CXCL1* (A) and *CXCL2* (B). *p-*values were calculated using the log-rank test. Data were derived from RNA-seq and obtained from the PRECOG (128-131), and TIDE (110, 112, 113).

**(C)** KM plots of OS for patients with breast cancer, grouped by high or low expression of CXCL*1* (top) and *CXCL2* (bottom). *p-*values were calculated using the log-rank test; Data derived from RNA-seq and obtained from the KM plotter website (114).

**(D)** Receiver Operating Characteristic (ROC) curves depicting the predictive value of *CXCL1* and *CXCL2* expression levels for OS in breast cancer patients. Data are consistent with (A-B) and derived from PRECOG (128-131) and TIDE (110, 112, 113).

**(E)** KM plots of OS for patients with melanoma (98) or glioblastoma (104) or bladder cancer (132) who received ICB therapy, grouped by high or low expression of *CXCL1* and *CXCL2*. *p*-values were determined using the log-rank test.

**(F)** Distribution of genetic alterations of *CXCL1* and *CXCL2* in TCGA breast cancer patients. Frequency (left) and counts (right) of alterations are shown.

**Fig. S2. Ponatinib inhibits 4T1 and MC38 tumor growth *in vivo***

**(A)** RT-qPCR analysis of LM2 (breast cancer), MCF7 (breast cancer), SUM159 (breast cancer) and SW620 (colon cancer) cells treated with 1 μmol/L ponatinib for 24 hours. Values were normalized to the vehicle (DMSO) control group values. Data are presented as means ± SD. Statistical significance was assessed by unpaired two-tailed Student’s *t*-test. **p* < 0.05, ***p* < 0.01 and *****p* < 0.0001.

**(B)** Schematic diagram for the *in vivo* drug studies in 4T1-tumor-bearing mice. Ponatinib (30 mg/kg) or vehicle was orally administered four days per week, starting when the tumor volume reached 100 mm³.

**(C)** Growth of 4T1 tumors in BALB/c WT mice treated with ponatinib or vehicle. Bar graph displaying the final tumor weight. Statistical significance was determined by unpaired two-tailed Student’s *t*-tests. ****p* < 0.001. An image of 4T1 tumors from the indicated groups is shown.

**(D)** Schematic diagram for the *in vivo* drug studies in MC38-bearing mice. Ponatinib (30 mg/kg) or vehicle was orally administered four days per week, starting when the tumor volume reached 200 mm³.

**(E)** Growth of MC38 tumors in C57BL/6 WT mice (n = 10 mice per group) treated with ponatinib or vehicle. Bar graph displaying the final tumor weight. Statistical significance was determined by unpaired two-tailed Student’s *t*-tests. ****p* < 0.001. An image of MC38 tumors from the indicated groups is shown.

Data are presented as means ± SEM.

**Fig. S3. SRC/ABL1/ABL2 are not the targets for the inhibition of *CXCL1* and *CXCL2* expression in TNBC cells by ponatinib**

**(A-B)** RT-qPCR analysis of knockdown efficiency **(A)** or *CXCL1* and *CXCL2* mRNA levels **(B)** in MDA-MB-231 cells.

The RT-qPCR data were normalized to *GAPDH*. Data represent mean ± SD. Statistical significance was calculated by unpaired two-tailed Student’s *t*-test. ns, *p* > 0.05, **p* < 0.05, ***p* < 0.01 and ****p* < 0.001.

**Fig. S4. Ponatinib downregulates *CXCL1* and *CXCL2* transcription through p38 dephosphorylation**

**(A-E)** Western blot analysis of the indicated proteins in multiple cancer cell lines treated with 1 μmol/L ponatinib or DMSO.

**Fig. S5. STAT1 loss-of-function suppresses the expression of *CXCL1* and *CXCL2***

**(A)** RT-qPCR analysis of *CXCL1* and *CXCL2* mRNA levels in MDA-MB-231 cells treated with the indicated concentrations of p38 inhibitors

**(B)** Western blot analysis shows the knockdown efficiency of STAT1 in MDA-MB-231 cells (top) or 4T1 cells (bottom).

**(C)** RT-qPCR analysis of *CXCL1* and *CXCL2* mRNA levels in MDA-MB-231 cells (left) or 4T1 cells (right) with *STAT1* knockdown compared with negative control. The RT-qPCR data were normalized to *GAPDH*.

**(D)** Correlation between *STAT1* expression and *CXCL1*/*CXCL2* expression in BRCA patients (67) from the CPTAC database. Each dot represents an individual patient, and *p*-values were calculated using Pearson's correlation test.

**(E)** SpatialFeaturePlots showing the spatial expression patterns of *CXCL1*, *CXCL2*, and *STAT1* in a breast cancer tissue section (68). Spatial transcriptomics was used to assess gene expression levels. Tumor cell enriched regions are highlighted with black shapes.

**(F and G)** Western blot analysis of the indicated proteins in MDA-MB-231 cells **(F)** or 4T1 cells **(G)** with knockdown of *SRC*, *ABL1*, or *ABL2,* compared to the negative control.

Data represent mean ± SD. Statistical significance was calculated by unpaired two-tailed Student’s *t*-test. **p* < 0.05 and ***p* < 0.01.

**Fig. S6. Higher MDSC frequencies are correlated to increased breast cancer progression**

**(A)** Cell types in the training scRNA-seq dataset from breast cancer with well-defined MDSC classification (71).

**(B-C).** Performance of the classifier generated using the scPred package with the training dataset (71).

**(D)** Correlation of MDSC infiltration level with *CXCL1* and *CXCL2* expression levels in breast cancer. Each dot represents a patient, and the Rhos were calculated using TIMER version 2 (72). *p-*values were determined using the Spearman correlation test, for note, for *CXCL1*, while a linear trend was not visually apparent, statistical analysis confirmed a significant positive correlation (*p* < 0.05, ρ > 0).

**(E-F)** Correlation between *CD33* expression and *CXCL1*/*CXCL2* expression in BRCA patients from the TCGA (72) and CPTAC databases (67). Each dot represents an individual patient.

**(G)** Protein levels of CXCL2 and CD33 were assessed by IHC in 129 TNBC tissue samples. Expression was quantified using the H-score method, and statistical significance was determined by Chi-square test.

**(H)** Representative IHC images showing CXCL2 and CD33 expression in TNBC tissues from four patients.

**Fig. S7. The expression of *CXCL1* and *CXCL2* in tumor cells is related to MDSC infiltration and an immunosuppressive TME**

**(A)** Expression of MDSC immunosuppressive genes (74, 75) in the breast cancer TME comparing patients with high or low expression of *CXCL1* and *CXCL2*. *p-*values were determined using the Mann-Whitney test.

**(B)** Correlation of *CXCL1* and *CXCL2* expression levels with fifteen MDSC immunosuppressive genes (74, 75) in breast cancer. The red bars represent positive correlation, and blue bars represent negative correlation. Data were calculated using the breast cancer scRNA-seq dataset (65).

**(C)** Correlation of *CXCL1* and *CXCL2* expression levels with four MDSC immunosuppressive genes (*IL1B*, *IL6*, *TREM1*, and *ARG2* (74, 75)) in breast cancer. Each dot represents a patient (65).

**(D)** Correlation of *CXCL1* and *CXCL2* expression levels with five MDSC immunosuppressive genes (*IL6*, *IL10*, *CD274*, *IDO1*, and *ADAM17* (74, 75)) in breast cancer. Each dot represents a patient. Rho and *p-*values were calculated using TIMER version 2. *p-*values were determined using the Spearman correlation test.

**(E-F)** Correlation of *CXCL1* **(E)** and *CXCL2* **(F)** expression levels with fifteen MDSC immunosuppressive genes (74, 75) across 40 TCGA cancer types. Red cells indicate a positive correlation, and blue cells indicate a negative correlation. Data were calculated using TIMER version 2 (72).

**(G)** Correlation of CD4^+^ T cell infiltration level with *CXCL1* and *CXCL2* expression levels in breast cancer. Each dot represents a patient, and Rhos were calculated using TIMER version 2 (72). *p-*values were determined using the Spearman correlation test.

**(H)** Correlation of *CXCL1* expression levels with *CD8A*, *CD8B*, and *GZMB* expression levels. Each dot represents a patient. Rho and *p-*values were calculated using TIMER version 2 (72). *p-*values were determined using the Spearman correlation test.

**(I)** Correlation of *CXCL1* or *CXCL2* expression levels on tumor cells with *IFNG*, *GZMB*, *GZMK*, *CD8B*, and *PRF1* expression levels on T cells in the TME of breast cancer. Each dot represents a patient, calculated with breast cancer scRNA-seq data (65).

**Fig. S8. Ponatinib slightly affects the distribution of immune cells in the spleen *in vivo***

**(A)** Flow cytometry analysis of MDSC in spleens from 4T1 tumor-bearing BALB/c WT mice treated with ponatinib or vehicle.

**(B)** Flow cytometry analysis of PMN-MDSC and M-MDSC in spleens from 4T1 tumor-bearing BALB/c WT mice treated with ponatinib or vehicle.

**(C)** Flow cytometry analysis of MDSC in spleens from 4T1 tumor-bearing BALB/c nude mice treated with ponatinib or vehicle.

**(D)** Gating strategy for flow cytometry analysis.

**(E)** Flow cytometry analysis of CD3^+^ T cells in spleens from 4T1 tumor-bearing BALB/c WT mice treated with ponatinib or vehicle.

**(F)** Flow cytometry analysis of CD4^+^ T cells and CD8^+^ T cells in spleens from 4T1 tumor-bearing BALB/c WT mice treated with ponatinib or vehicle.

**(G)** Flow cytometry analysis of NK cells (CD45^+^CD49b^+^) in spleens from 4T1 tumor-bearing BALB/c nude mice treated with ponatinib or vehicle.

Data are presented as means ± SEM. Statistical significance was determined by unpaired two-tailed Student’s *t*-tests. ns, *p* > 0.05, **p* < 0.05.

**Fig. S9. The expression levels of *CXCL1* and *CXCL2* predict the response to immunotherapy**

**(A)** Expression of *CXCL1* and *CXCL2* in the breast cancer TME, comparing Es with NEs. Each dot represents a patient. *p-*values were determined using the Mann–Whitney test.

**(B)** Expression of *CXCL1* and *CXCL2* in tumor cells, comparing Es with NEs, calculated using the breast cancer scRNA-seq dataset (65). *p-*values were determined using the Mann–Whitney test.

**(C)** Average expression of *CXCL1* and *CXCL2* comparing responder (R) with non-responder (NR) to ICB treatment, shown for four separate datasets (left four panels) and in merged form (right). Data represent ICB response for patients across four datasets (97-99, 109). *p-*values were determined using the Mann–Whitney test.

**(D-E)** Area Under the Curve (AUC) for the performance of signatures in predicting ICB response across nine datasets (Table S1**)**, represented as merged **(D)** or separated **(E)**. AUC in **(D)** represents the average AUC across the nine datasets; the performance of a random predictor (AUC = 0.5) is represented by the dashed line. TMB, tumor mutation burden; MSI, microsatellite instability signature.

**(F)** ICB response and a signature of known MDSC-related gene expression (71, 74, 75, 79, 80) in breast cancer (65, 71). Cells are colored by ICB response group (left) and by MDSC gene expression level (right). MDSCs were determined in the breast cancer scRNA-seq dataset, including 29 TNBC patients who received ICB therapy (EGAD00001006608) (65), through learning from another breast cancer scRNA-seq dataset with MDSC well-defined (GSE139125) (71), using the scPred package (70) for cell type prediction.

**Table S1. Information on datasets used in this study**

**Table S2. The results of molecular docking**

**Table S3. Clinical and pathological information for 129 TNBC patients included in the tissue microarray used for IHC staining**
